# Correlations between long inverted repeat (LIR) features, deletion size and distance from breakpoint in human gross gene deletions

**DOI:** 10.1038/srep08300

**Published:** 2015-02-06

**Authors:** Nevim Aygun

**Affiliations:** 1 Department of Medical Biology, Faculty of Medicine, Dokuz Eylul University, Inciralti, Izmir, Turkey

## Abstract

Long inverted repeats (LIRs) have been shown to induce genomic deletions in yeast. In this study, LIRs were investigated within ±10 kb spanning each breakpoint from 109 human gross deletions, using Inverted Repeat Finder (IRF) software. LIR number was significantly higher at the breakpoint regions, than in control segments (*P* < 0.001). In addition, it was found that strong correlation between 5′ and 3′ LIR numbers, suggesting contribution to DNA sequence evolution (*r* = 0.85, *P* < 0.001). 138 LIR features at ±3 kb breakpoints in 89 (81%) of 109 gross deletions were evaluated. Significant correlations were found between distance from breakpoint and loop length (*r* = −0.18, *P* < 0.05) and stem length (*r* = −0.18, *P* < 0.05), suggesting DNA strands are potentially broken in locations closer to bigger LIRs. In addition, bigger loops cause larger deletions (*r* = 0.19, *P* < 0.05). Moreover, loop length (*r* = 0.29, *P* < 0.02) and identity between stem copies (*r* = 0.30, *P* < 0.05) of 3′ LIRs were more important in larger deletions. Consequently, DNA breaks may form via LIR-induced cruciform structure during replication. DNA ends may be later repaired by non-homologous end-joining (NHEJ), with following deletion.

Long inverted repeats (LIRs) are imperfect or near to perfect repetitive DNA sequence elements that can form secondary stem-loop structures in prokaryotic and eukaryotic genomes^1^,^2^,^3^. LIRs may induce stem loops through matching complementary repeats placed in inverted orientation, convertible to the hairpins in single stranded DNA or cruciforms in double stranded DNA^4^,^5^. It was found that LIRs involved deletion and recombination events in yeast *Saccharomyces cerevisiae*^6^,^7^.

Gross gene deletions are genomic rearrangements that can be observed in many types of human cancers and inherited diseases^8^,^9^,^10^,^11^,^12^,^13^. Deletion and duplication mutations can vary in size from thousands to hundreds of thousands of base pairs in length in the human genome^14^. It has been proposed that three major mechanisms are responsible for genomic rearrangements, including human genome deletions^15^. They are non-allelic homologous recombination (NAHR), non-homologous end-joining (NHEJ), and fork stalling and template switching (FoSTeS) models. Some genomic rearrangements are recurrent, with a common size and fixed breakpoints between low copy repeats (LCRs). Recurrent rearrangements are mostly mediated by NAHR between two LCRs^16^. Conversely, non-recurrent rearrangements have different sizes and distinct breakpoints in each event, and are performed by NHEJ and FoSTeS models^15^. Gu et al. suggests that the FoSTeS model is a replication-based rearrangement pathway that may operate over long distances (from 120 to 550 kb) through template switching^15^. Alternatively, it has been proposed that palindrome or cruciform structures may stimulate the FoSTeS model^15^.

Breakpoints of gross gene deletions coincide with non-B DNA conformations, including hairpin/cruciform structures^17^. Hairpins are reported to form by direct repeats^18^. Direct repeats have ranges from 2 to 8 bp, and are associated with small deletion breakpoints in human genetic diseases^19^. Moreover, retinoblastoma gene deletion involves direct repeats within the deletion breakpoints^20^.

Short direct repeats were also detected in 15 proximal breakpoints of the dystrophine gene, which has large deletions^21^. Short IRs and IR inversions were found in 83% of deletions + small insertions, while short direct repeats were detected only in simple deletion breakpoints^22^.

Two highly homologous *Alu* repeats in inverted orientations were found in the vicinity of gross deletion breakpoints in the von Willebrand factor (*VWF*) gene^23^. Furthermore, LINEs, LTR repetitive elements, and SINEs (including *Alus*), were enriched at breakpoints of rare pathogenic microdeletions^24^. Vissers et al. also suggests that microhomology levels of breakpoint junctions play an important role in replication-based mechanisms, such as FoSTeS and microhomology-mediated break-induced replication (MMBIR)^24^. Zhang et al. also suggested replication fork stalling to initiate FoSTeS^25^.

Gordenin et al. showed that LIRs cause deletion in *Saccharomyces cerevisiae*^6^. Lobachev et al. suggested they form stem-like secondary structures on single stranded DNA during replication, thereby causing deletions^26^. Warburton et al. found that some IRs are capable of transforming into cruciform structures, with intrastrand double helices, termed stems and unpaired loops forming internal spacers^1^. The four-way junction of this suggested IR pattern is similar to the Holliday structure. Eichman et al. showed formation of the Holliday junction in synthetic IR DNA using X-ray crystallography^27^. From this work, it was proposed that IRs may be involved in homologous recombination. Bacolla and Wells indicated that IRs may form cruciform structures, and are often found at genomic rearrangement breakpoints^18^.

Genomes of many complex organisms have been investigated for larger IRs. It was determined that higher eukaryotic genomes include many imperfect and near-to-perfect LIRs^28^,^29^,^30^,^31^. In mice, a perfect LIR was shown to create a large deletion^32^. Subsequently, it was decided that criteria for LIRs in genomic rearrangements involved recombination. In this regards, Wang and Leung reported that LIRs with stem length >30 bp, identity between stem copies (hereafter stem identity) >85% and internal spacer of <2 kb, are recombinogenic in genomes of humans and some other organisms^2^. Voineagu et al. demonstrated that *Alu* IRs with 100% sequence homology of stem copies, triggers strong replication blockage^3^. However, *Alu* IRs with 75% stem identity between repetitive halves caused mild replication blockage in *E. coli* cells^3^.

Potential models referred to as replication slippage and hairpin nicking were proposed by Akgün et al. to explain the mechanism underlying LIR induced deletions^4^. With these models, many deletions formed inside palindrome stems or loops are explained. However, alternative models are required for clarifying the mechanisms of larger deletions formed in close proximity to palindromes. To understand how gross gene deletions occur in human cancers and inherited diseases, this present study investigated the significance of LIRs on breakpoint regions of human gross gene deletions.

## Results

### Identification of long inverted repeats in breakpoint regions of gross gene deletions

**Figure 1:**
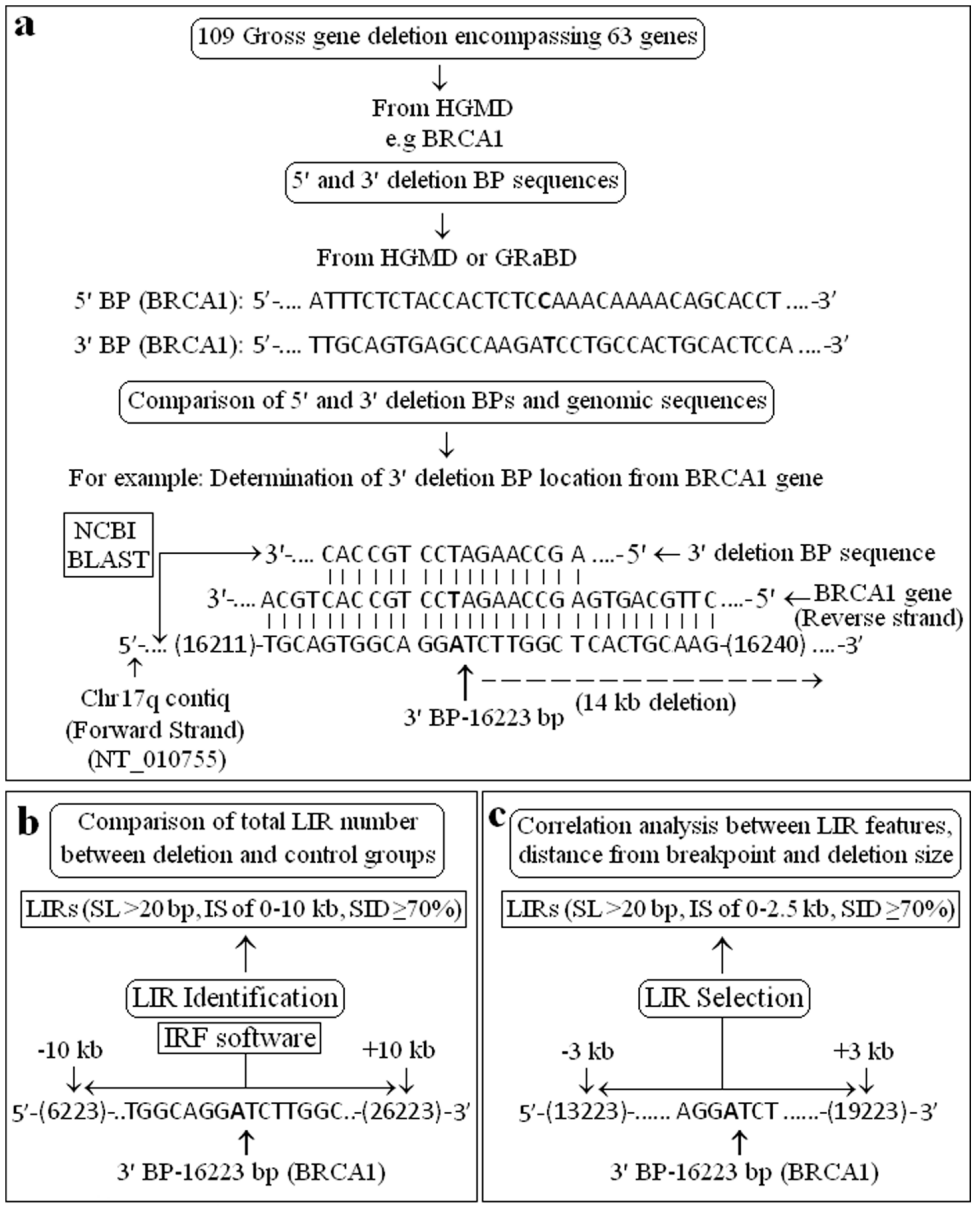
LIR identification and selection were performed in 218 genomic regions including 5′ and 3′ breakpoints from 109 gross deletions involving 63 human genes using IRF software. (a) DNA sequences from 5′ and 3′ deletion BPs were obtained from HGMD and GRaBD. Each BP-DNA sequence and corresponding gene was compaired using NCBI BLAST. Deletion BP locations were determined in related genes. 3′ BP sequence from *BRCA1* gene was presented for describing BLAST comparing process. (b) LIR identification was done within ±10 kb flanking sequences each of 5′ and 3′ deletion BPs and 20 kb DNA fragments from control groups using IRF. LIRs with SL > 20 bp, IS of 0–10 kb, SID ≥ 70% were included for comparing total LIR number between deletion and control groups using Mann Whitney *U* test. (c) LIR selection was made within ±3 kb flanking sequences each of 5′ and 3′ BPs in deletion group. LIRs with SL > 20 bp, IS of 0–2.5 kb, SID ≥ 70% were selected for analysing of correlations between LIR features, distance from breakpoint and deletion size using Pearson's coefficient. Abbreviations: Bp, base pair; BP, breakpoint; GRaBD, gross rearrangement breakpoint database; HGMD, human gene mutation database; IRF, inverted repeat finder; IS, internal spacer; kb, kilobase; LIR, long inverted repeat; SID, stem identity; SL, stem length.

Sequences from 218 breakpoint regions of 63 gross gene deletions were taken from references 33,34,35,36,37,38,39,40,41,42,43,44,45,46,47,48,49,50,51,52,53,54,55,56,57,58,59,60,61,62,63,64,65,66,67,68,69,70,71,72,73,74,75,76,77,78,79,80,81,82,83,84,85,86,87,88,89 (see Supplementary Table S1 online) listed in the HGMD^90^,^91^ and GRaBD^92^,^93^ (Figure 1a). LIRs with stem length >20 bp on surrounding (±10 kb) each breakpoint were investigated using IRF^94^,^95^ (Figure 1b). In total, 218 genomic regions, including 5′ and 3′ breakpoints from 109 gross deletions involving 63 different genes (Table 1), were analysed. Total number of LIRs was determined within ±10 kb regions flanking each breakpoint. In the deletion group, a total of 2723 LIRs were detected (see Supplementary Table S2 online). A total of 1345 LIRs were also identified in 20 kb segments from 220 control sequences (see Supplementary Table S2 online).

Mean ranks of LIR numbers were compared between gross deletion breakpoints and control sequences using the Mann Whitney *U* test.The mean LIR number was significantly higher at the breakpoint regions from gross gene deletions, than in control group (*P* < 0.001).

In addition, associations between 5′ and 3′ LIR numbers within ±10 kb regions flanking each breakpoint were determined using Pearson's correlation coefficients. Positive, strongly significant association was found between LIR numbers from 5′ and 3′ breakpoints in 109 gross deletions (*r* = 0.85, *P* < 0.001).

Additionally, Spearman's correlation showed that a negative moderately significant associations were found between deletion size and 5′ LIR number (*r_s_* = −0.30, *P* < 0.003), and 3′ LIR number (*r_s_* = −0.30, *P* < 0.002) in 109 gross deletions respectively.

### Features of LIRs selected within ±3 kb genomic regions flanking 5′ and 3′ deletion breakpoints

**Figure 2:**
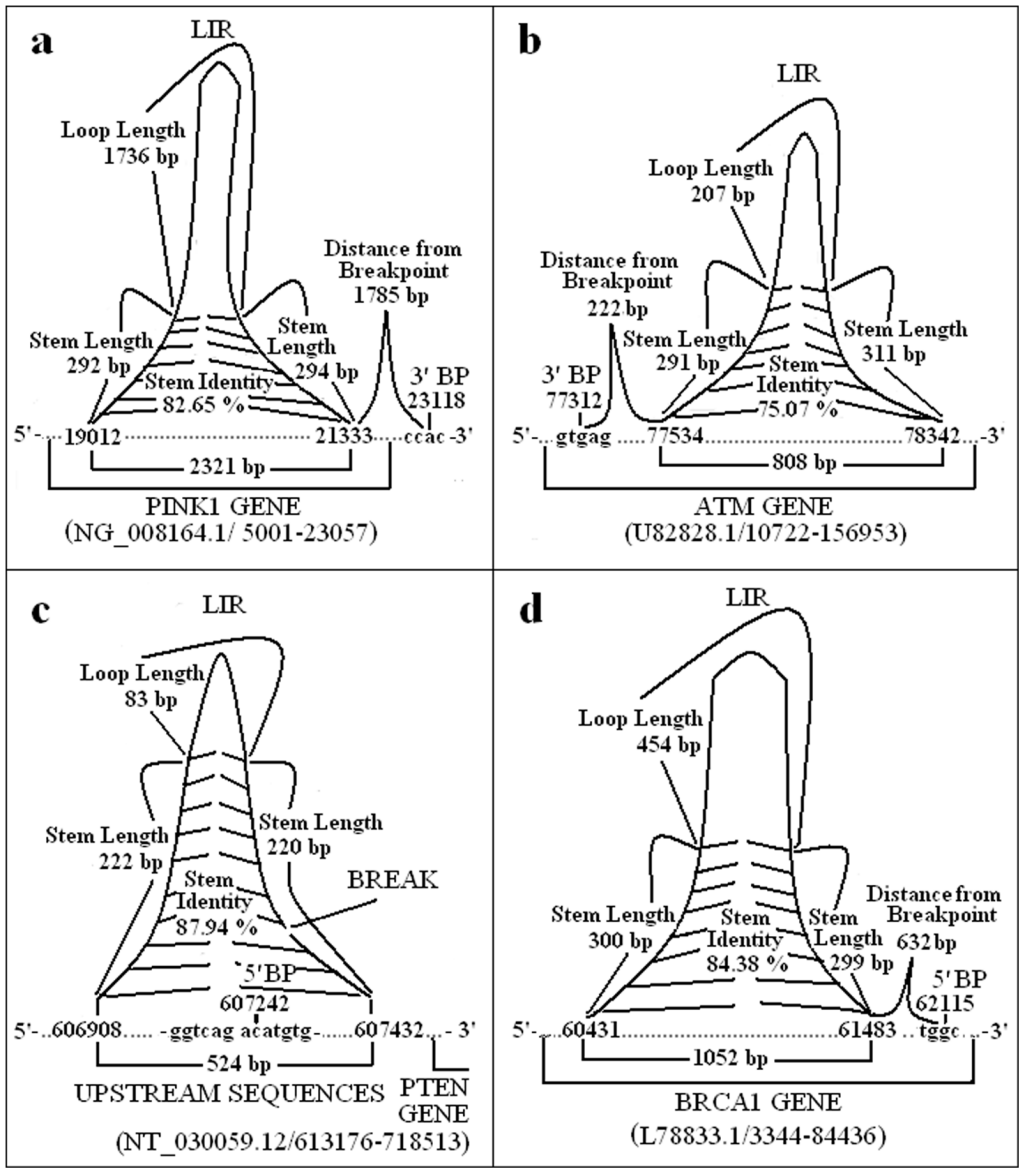
Breakpoint regions of *PINK1*, *ATM*, *PTEN* and *BRCA1* genes. Sizes of LIR features, e.g. stem length, stem identity and internal spacer (loop length) are shown. NCBI accession numbers of each gene are provided. Coordinates correspond to GenBank sequences. (a) 3′ breakpoint sequence of the *PINK1* deletion is in the downstream of gene. The LIR of *PINK1* is located at the upstream of 1785 bp from 3′ breakpoint, has a stem length of 292 bp, internal spacer of 1736 bp, and stem identity of 82.65%. (b) 3′ breakpoint sequence of the *ATM* deletion is within the gene. The LIR of *ATM* is located at the 222 bp downstream of 3′ breakpoint, and has a stem length of 291 bp, internal spacer of 207 bp, and stem identity of 75.07%. (c) 5′ breakpoint sequence of the *PTEN* deletion is in the upstream of gene. The LIR of *PTEN* includes 5′ breakpoint, and has a stem length of 220 bp, internal spacer of 83 bp, and stem identity of 87.94%. (d) 5′ breakpoint sequence of the *BRCA1* deletion is within the gene. The LIR of *BRCA1* is located at the upstream of 632 bp from 5′ breakpoint, and has a stem length of 299 bp, internal spacer of 454 bp, and stem identity of 84.38%. Abbreviation: Bp, base pair; BP, breakpoint; LIR, long inverted repeat.

Next, LIRs were selected using appropriate criteria (outlined in Materials and Methods) (Figure 1c). Properties of these selected LIRs from 5′ and 3′ breakpoints were analysed. In total, 138 LIRs at distance of 0–3 kb from breakpoints, with stem length >20 bp, internal spacer of 0–2.5 kb, and stem identity >70% were detected (see Supplementary Table S3 online). The stem lengths and identities, internal spacer (loop) lengths and distances from breakpoints of LIRs were determined at the breakpoint locations of genes involved in gross deletions, including *PINK1* (NG_008164.1; 5001-23057), *ATM* (U82828.1; 10722-156953), *PTEN* (NT_030059.12; 613176-718513) and *BRCA1* (L78833.1; 3344-84436) (Figure 2).

### Distribution of LIRs at the 5′ and 3′ breakpoint regions from gross gene deletions

**Figure 3:**
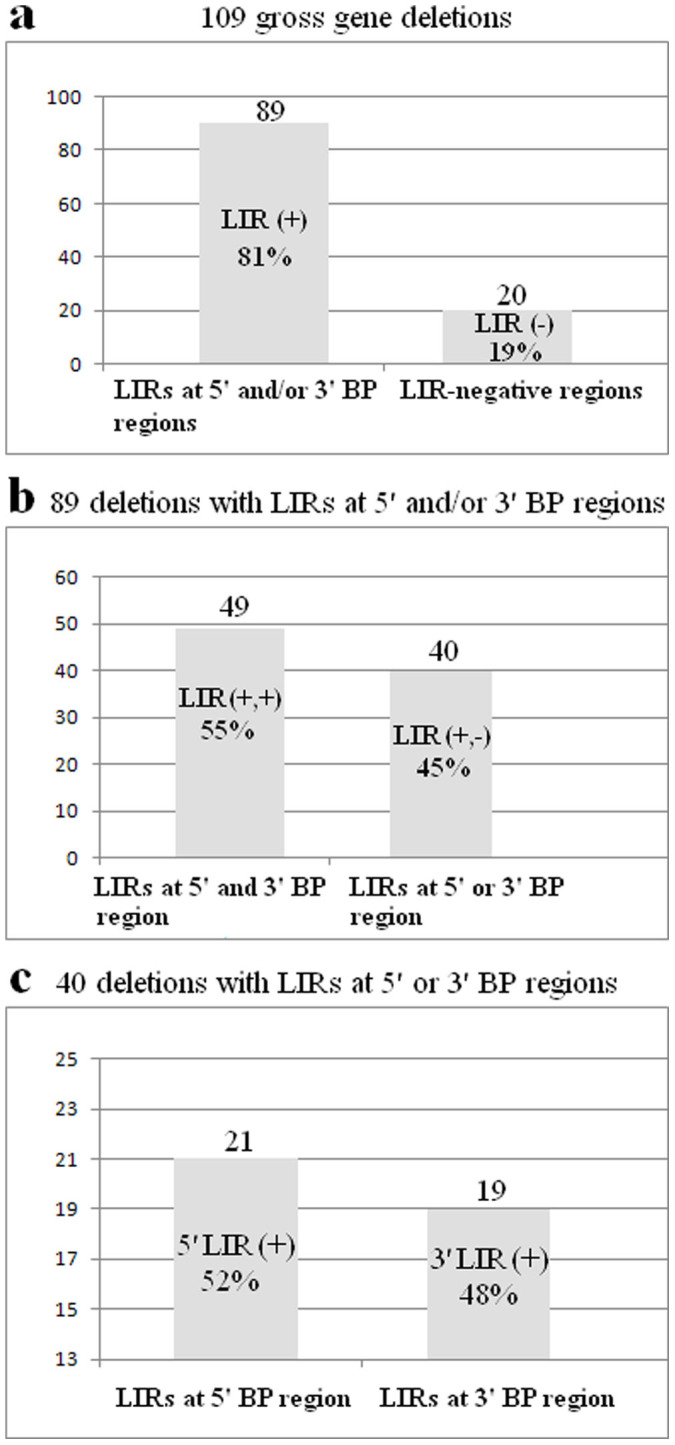
Distribution of 138 LIRs at the 5′ and 3′ BP regions of 109 gross gene deletions. (a) LIRs were detected in 89 (81%) gene deletions. (b) In 49 of these deletions, LIRs were located at both 5′ and 3′ BP regions. (c) Among the 40 deletions with LIRs at one of the breakpoint regions, in 21, the LIRs were at the 5′ BP region, and in 19 deletions, at the 3′ BP region. Abbreviations: BP, breakpoint; LIR, long inverted repeat.

LIR distribution was also examined at the 218 genomic locations, including 5′ and 3′ breakpoint regions from 109 gross gene deletions (see Supplementary Figure S1 online). From these 218 locations, 138 LIRs were detected at the 5′ and/or 3′ breakpoints from 89 gross gene deletions (Figure 3a). Moreover, 49 of 89 gross deletions had 98 LIRs in both 5′ and 3′ deletion breakpoints (Fig. 3b), and 40 of 89 gross deletion had LIR at the 5′ or 3′ breakpoint sites (Figure 3c).

### Correlations between LIR features such as length and identity of stem, internal spacer length and distance from breakpoint, and also deletion size in 89 gross gene deletions

In all identified 138 LIRs, at the 5′ and/or 3′ breakpoints in 89 (81%) of 109 gross deletions had stem lengths between 24 and 973 bp (see Supplementary Fig. S2 online), with stem identities between 70.54 and 100% (see Supplementary Figure S3 online). These LIRs were located at the distance of ±0–2,539 bp from 5′ and 3′ breakpoints (see Supplementary Fig. S4 online), with internal spacer lengths between 0 and 2,435 bp (see Supplementary Figure S5 online).

Associations between features of these LIRs were examined using Pearson's correlation coefficient. Low to moderately significant correlations were found between certain LIR features (e.g. stem length and identity, internal spacer length and distance from breakpoint). In all 138 LIRs located at the regions including 5′ and 3′ breakpoints, negative correlations were found between stem length and stem identity (*r* = −0.49, *P* < 0.001), internal spacer length and distance from breakpoint (*r* = −0.18, *P* < 0.05), stem length and distance from breakpoint (*r* = −0.18, *P* < 0.05), and internal spacer length and stem identity (*r* = −0.17, *P* < 0.05). Conversely, a moderately positive correlation was found between internal spacer length and stem length (*r* = 0.27, *P* < 0.002). No correlation was found between stem identity and distance from breakpoint (*r* = −0.008, *P* > 0.1).

Moreover, associations between gross gene deletion size and features of the 138 LIRs were analysed by Pearson's correlation coefficient. It was found that positive significant correlation between internal spacer length and deletion size (*r* = 0.19, *P* < 0.05). However, no correlations were found between deletion size and three other LIR features, specifically, stem length (*r* = 0.01, *P* > 0.1), stem identity (*r* = −0.06, *P* > 0.1), and distance from breakpoint (*r* = 0.08, *P* > 0.1).

In addition, 5′ and 3′ LIR features from 89 gross deletions were re-examined individually. Thus, associations between properties of 70 and 68 LIRs located on 5′ and 3′ breakpoints, respectively, and deletion size, were analysed by Pearson's coefficient. Negative moderate to strong correlations were found between internal spacer length and stem identity (*r* = −0.28, *P* < 0.02), and stem length and stem identity (*r* = −0.57, *P* < 0.001) for LIRs within 5′ breakpoints.

A positive moderate correlation was found between internal spacer length and stem length (*r* = 0.35, *P* < 0.004) for LIRs within 3′ breakpoints. In addition, negative moderate correlations were found between stem identity and stem length (*r* = −0.40, *P* < 0.002), and distance from breakpoint and stem length (*r* = −0.31, *P* < 0.02).

Furthermore, positive moderately significant correlation was found between internal spacer length of 3′ LIRs and deletion size (*r* = 0.29, *P* < 0.02). However, no correlation was found between internal spacer length of 5′ LIRs and deletion size (*r* = −0.16, *P* > 0.1). No correlations were found between deletion size and three other LIR features, specifically, stem length (5′: *r* = −0.06, *P* > 0.1; 3′: *r* = 0.04, *P* > 0.1), stem identity (5′: *r* = 0.12, *P* > 0.1; 3′: *r* = −0.10, *P* > 0.1), and distance from breakpoint (5′: *r* = 0.22, *P* > 0.05; 3′: *r* = 0.08, *P* > 0.1).

### Correlations between stem length and identity, internal spacer length and distance from breakpoint, and also deletion size in 49 gross gene deletions including LIRs at both of 5′ and 3′ breakpoints

In addition, 98 LIRs were identified in both 5′ and 3′ breakpoints from 49 of 89 gross gene deletions (Figure 3b). These LIRs had stem identities of 71.65–100%, stem lengths of 27–603 bp, and internal spacer lengths of 0–2,435 bp (see Supplementary Table S3 and Figure S6 online). Features of these 98 LIRs were analysed using Pearson's correlation coefficient. Low to moderately significant correlations were found between certain LIR features, including stem length, stem identity, loop length and distance from breakpoint. Positive correlation was found between internal spacer length and stem length (*r* = 0.23, *P* < 0.05). Negative moderate correlations were found between stem identity and stem length (*r* = −0.39, *P* < 0.001), and distance from breakpoint and stem length (*r* = −0.31, *P* < 0.003). However, no correlations were found between internal spacer length and stem identity (*r* = −0.08, *P* > 0.1), internal spacer length and distance from breakpoint (*r* = −0.13, *P* > 0.1), and stem identity and distance from breakpoint (*r* = 0.06, *P* > 0.1).

Furthermore, 5′ and 3′ breakpoint regions of these 98 LIRs were examined individually. Associations between LIR features from 5′ and 3′ breakpoint locations and gross gene deletion size in 49 gross deletions were analysed by Pearson's correlation method. A negative moderate correlation was found between stem length and distance from breakpoint for LIRs in 5′ breakpoint regions (*r* = −0.30, *P* < 0.05). Negative moderate correlation was also found between stem length and distance from breakpoint for LIRs in 3′ breakpoint regions (*r* = −0.33, *P* < 0.05). Strong negative correlation was found between stem length and stem identity from 3′ LIRs (*r* = −0.51, *P* < 0.001). Positive moderate correlation was found between stem length and internal spacer length from 3′ LIRs (*r* = 0.36, *P* < 0.02).

In addition, the relationship between 5′ and 3′ LIRs were analysed. Positive moderate correlation was found between distance from breakpoint for 5′ LIRs and stem identity of 3′ LIRs, involving 49 gross gene deletion regions (*r* = 0.28, *P* < 0.05).

Associations between deletion size and 5′ and 3′ LIR features from these 49 gross deletions were also analysed by Pearson's correlation method. Negative moderate correlation was found between stem identity of 5′ LIRs and deletion size (*r* = −0.40, *P* < 0.005). Positive moderate correlation was found between stem identity of 3′ LIRs and deletion size (*r* = 0.30, *P* < 0.05). However, no correlations were found between deletion size and loop length (5′: *r* = −0.04, *P* > 0.1; 3′: *r* = 0.04, *P* > 0.1), stem length (5′: *r* = 0.17, *P* > 0.1; 3′: *r* = −0.18, *P* > 0.1), and distance from breakpoint (5′: *r* = 0.11, *P* > 0.1; 3′: *r* = −0.10, *P* > 0.1).

### Correlations between length and identity of stem, internal spacer length and deletion size in 40 gross gene deletions including LIRs spanning 5′ or 3′ breakpoints

LIRs were also identified at the other 40 gross gene deletions containing LIRs in 5′ or 3′ breakpoints. In total, 21 and 19 of the 40 LIRs from 5′ and 3′ breakpoint sites, respectively, were analysed (Figure 3c). LIRs had stem identities of 70.54–100%, stem lengths of 24–973 bp, and internal spacer lengths of 0–2,422 bp, and were located at the distance of 0–2,311 bp from breakpoints (see Supplementary Table S3 online).

Associations between LIR features were analysed by Pearson's correlation coefficient. Moderate to strong significant correlations were found between LIR features, including stem length and stem identity, and internal spacer length and distance from breakpoint. In 40 LIRs, a positive moderate correlation was found between internal spacer length and stem length (*r* = 0.34, *P* < 0.05). In addition, negative correlations were found between internal spacer length and stem identity (*r* = −0.33, *P* < 0.05), and stem length and stem identity (*r* = −0.61, *P* < 0.001). However, no correlations were found between distance from breakpoint and internal spacer length (*r* = −0.25, *P* > 0.1), stem length (*r* = −0.02, *P* > 0.1), or stem identity (*r* = −0.12, *P* > 0.1).

Deletion size and LIR features were also analysed by Pearson's correlation method. A positive moderate correlation was found between internal spacer length of LIRs and deletion size (*r* = 0.35, *P* < 0.05). However, no correlations were found between deletion size and three other LIR features, specifically, stem length (*r* = 0.00, *P* > 0.1), stem identity (*r* = −0.08, *P* > 0.1), and distance from breakpoint (*r* = 0.09, *P* > 0.1).

### Re-examination of LIRs detected in only one of regions spanning 5′ and 3′ breakpoints of gross gene deletions

The 40 gross gene deletions containing LIRs in only one of genomic regions spanning 5′ and 3′ breakpoints were re-examined in terms of their ability to form new LIRs between breakpoints with LIRs and non LIRs in related deletion regions. LIRs with distance of 0–10 kb from breakpoints, stem identity >70% and stem length >150 bp were analysed.

**Figure 4:**
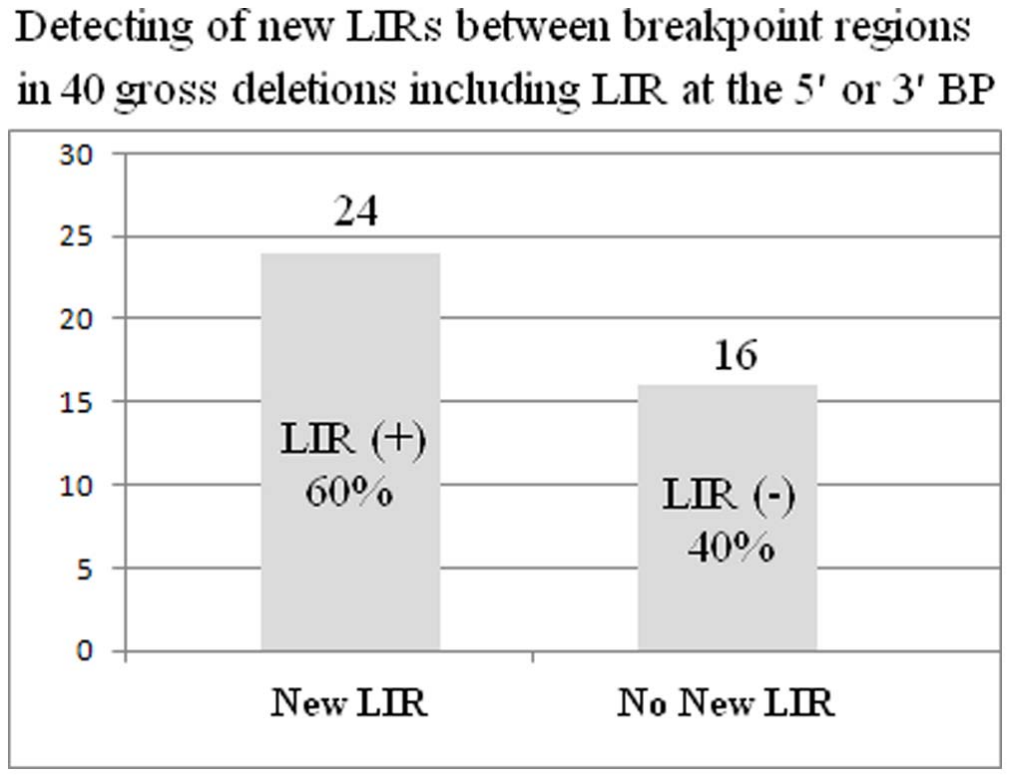
Identification of new LIRs between breakpoint regions of gross gene deletions including LIR at only one of the 5′ and 3′ BPs. New LIRs were detected between genomic sequences flanking breakpoints in 24 of the 40 gross deletions including LIR at the 5′ or 3′ BP. Abbreviations: BP, breakpoint; LIR, long inverted repeat.

**Figure 5:**
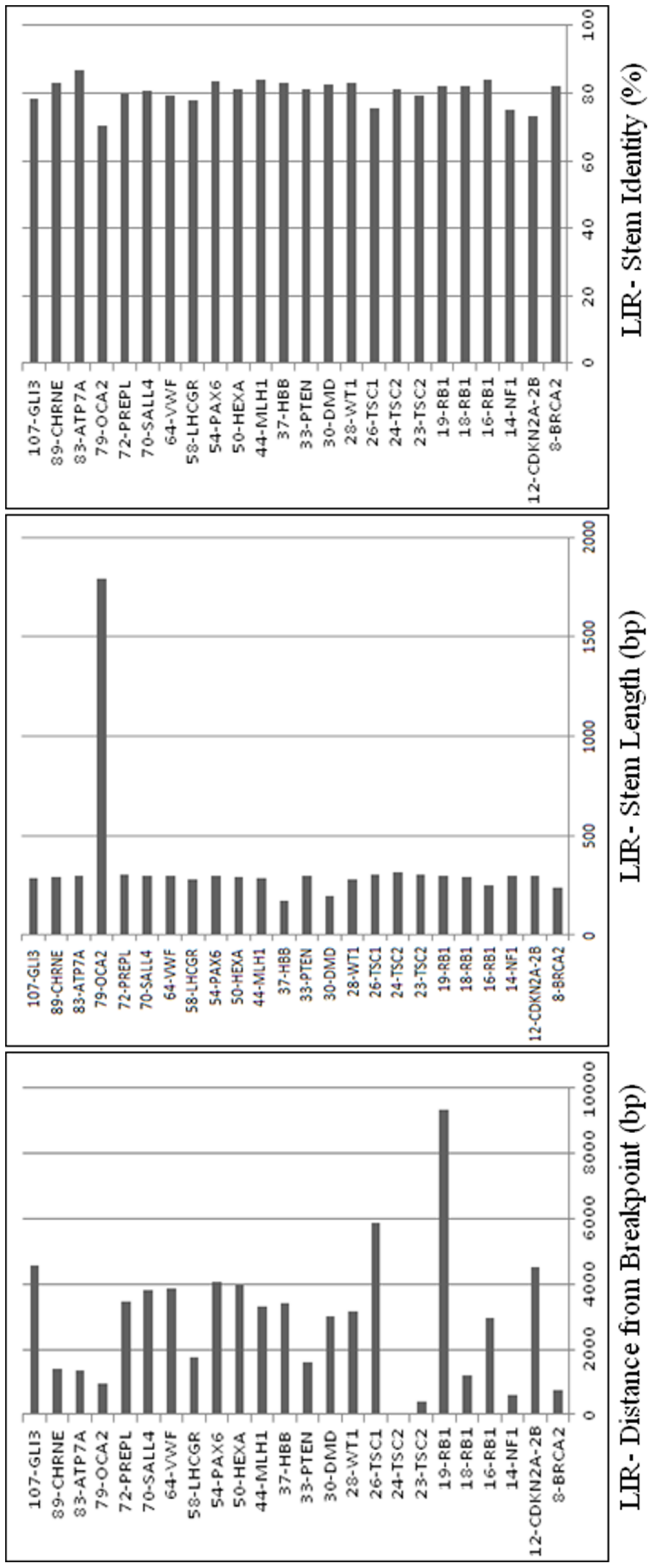
In 24 gross deletion, new identified LIRs with stem identity, stem length and distance from breakpoint were shown. Black bars indicate stem length, stem identity and distance from breakpoint of LIRs found between distant sites. Abbreviations: Bp, base pair; LIR, long inverted repeat.

In 24 of the 40 gross deletions, new LIRs between 5- and 10-kb genomic segments from 5′ and 3′ breakpoints containing LIR or no LIR, respectively, were found (see Supplementary Table S4 online; Figure 4). From these 24 gross deletions, LIR stem identities and lengths were determined to be 70.19–86.66% and 173–1789 bp, respectively (Figure 5). In addition, these LIRs were located at distance of ±42–9,330 bp from breakpoints (Figure 5).

Features of these 24 LIRs were analysed by Spearman's correlation method. A strong significantly negative correlation was found between stem length and stem identity (*r_s_* = −0.51, *P* < 0.02). No correlations were found between distance from breakpoint and stem length (*r_s_* = −0.08, *P* > 0.1) or stem identity (*r_s_* = −0.08, *P* > 0.1).

## Discussion

Deletion breakpoints are often associated with *Alu* and non-B DNA-forming elements such as short direct and inverted repeats, and inversions of inverted repeats in human genomic rearrangements^17^,^19^,^20^,^21^,^22^,^23^. In this study, LIRs within ±10 kb regions flanking 218 breakpoint sequences from gross gene deletions in human cancers and inherited diseases, were investigated by using IRF^94^,^95^ software. As a program that uses an algorithm presented by Benson^95^, IRF software can efficiently detect two or more contiguous approximate inverted repeats in sizes up to 700 kb at the same location on DNA sequences without the need to specify either the pattern or pattern size. In this way, IRF software served that present study accurately analyzes significance of relationship between LIR numbers and breakpoint regions in human gross gene deletions.

This work showed that the mean LIR number was significantly higher at the breakpoint regions of gross gene deletions, than in control group (*P* < 0.001). In addition, strongly significant positive correlation was found between 5′ and 3′ LIR numbers from breakpoint regions (*r* = 0.85, *P* < 0.001). In this regards, increasing LIR numbers can cause or induce chromosomal rearrangements (including duplication, recombination and/or deletion) in human genome during evolutionary process.

Furthermore, negative moderately significant associations were found between deletion size and 5′ and 3′ LIR numbers (*r_s_* = −0.30, *P* < 0.003; *r_s_* = −0.30, *P* < 0.002) in 109 gross deletions, respectively. This result indicates that increasing 5′ or 3′ LIR numbers at the breakpoints cause smaller deletion sizes. Over-LIR intensity may impede efficiency, strengthens and further kinetic properties of inverted repeats because of competing LIRs with each other. Consequently, these findings suggest that DNA sequence evolution may also be prosecuted by LIRs in human genome.

In *Saccharomyces cerevisiae*, Saini et al. reported that IRs induce mutagenesis by break formation at distant sites (up to 8 kb)^96^. Similarly, Lobachev et al. suggested that LIRs may stimulate recombination and deletion by forming secondary structures on the single strand DNA during replication^26^. In addition, Bacolla and Wells indicated that repetitive DNA motifs may fold into non-B DNA structures including cruciforms/hairpins, leading to genomic rearrangements associated with neurodegenerative and genomic disorders^18^.

In 138 LIRs identified in 89 gross deletion, significant associations were found between internal spacer length and distance from breakpoint (*r* = −0.18, *P* < 0.05), stem length and distance from breakpoint (*r* = −0.18, *P* < 0.05). These associations suggest DNA strand breaks potentially in locations close to larger LIRs. Similarly, Lobachev et al. reported that stimulation of deletions was positively correlated with IR size^26^. In addition, Lim et al. reported that IRs ≥ 800 bp are required for gene deletion effectiveness in *Saccharomyces cerevisiae*, showing IRs improve gene deletion efficiency up to 1.2 kb^97^.

In addition, a positive significant correlation between internal spacer length and deletion size in 138 LIRs was found (*r* = 0.19, *P* < 0.05), suggesting LIRs with bigger loops cause larger deletions at fragile DNA sites. Weiss and Wilson reported that loops with 25–247 nucleotides (nt) were efficiently and accurately repaired during homologous recombination^98^. It was suggested that bigger loops (>247 nt) cannot repair and excise in homologous recombination accurately, therefore cells with these loops may be subject to either apoptosis or NHEJ. If cells cannot induce apoptosis, it was suggested that LIRs > 247 nt may break DNA, and be repaired by NHEJ.

**Figure 6:**
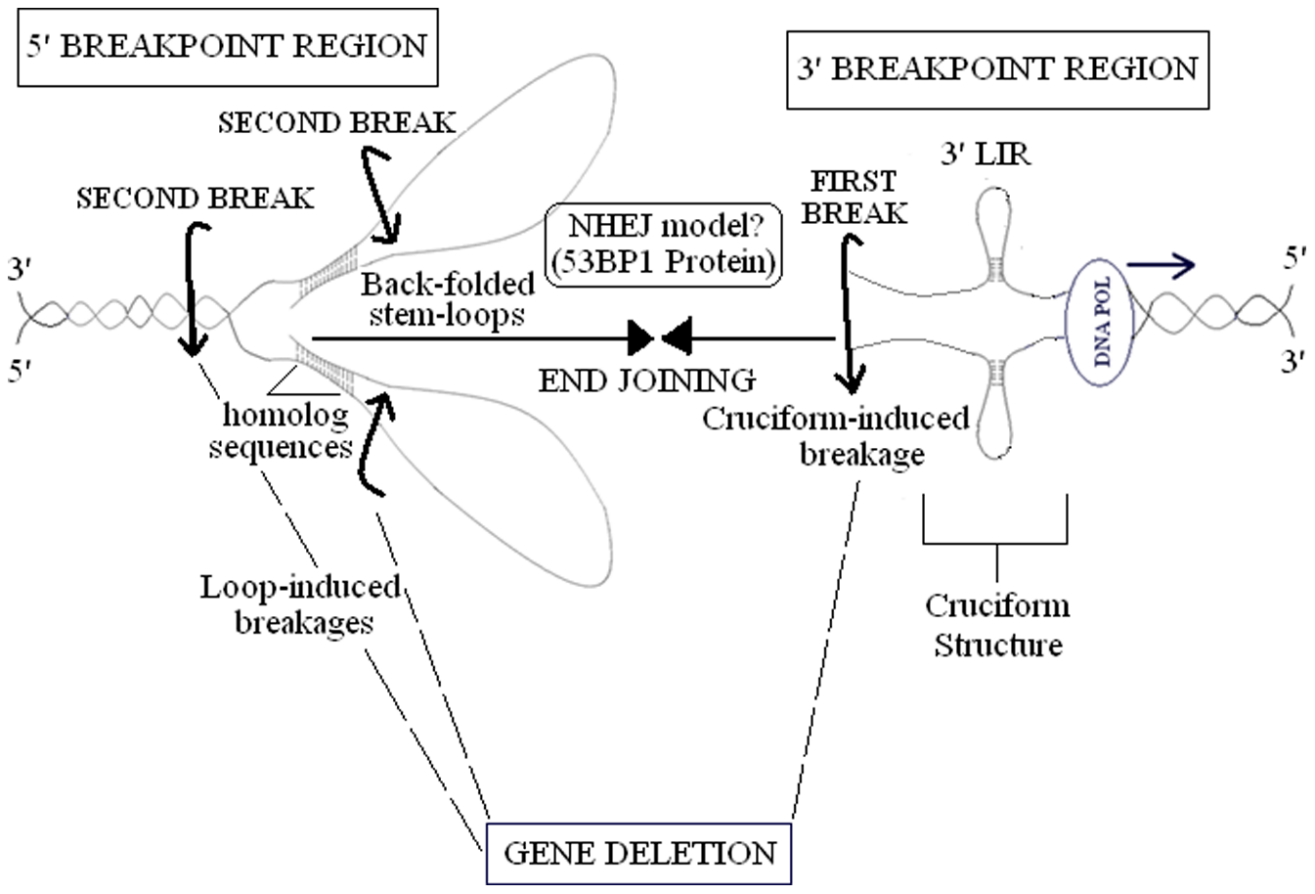
A model mechanism for single LIR-mediated gene deletion. LIR forming cruciform structure in the single strand DNA nearing 3′ breakpoint of the gross gene deletion during replication is shown. After the first break is occurred in the vicinity of 3′ LIR, second break is induced by back-folded stem-loop structures forming with homolog sequences between distant 5′ and 3′ breakpoint sites. Free DNA ends may combine via 53BP1-mediated NHEJ. Abbreviations: LIR, long inverted repeat; NHEJ, non-homologous end-joining.

In conclusion, larger deletions may more efficiently form by LIRs with larger loops at 5′ or 3′ breakpoints in human cancers and inherited diseases. DNA end may gain further kinetic properties, and match with distant brekpoint site (Figure 6).

Moreover, correlation between distance from breakpoint and stem length (*r* = −0.31, *P* < 0.02) was observed in 3′ LIRs from 89 gross deletions. These data suggest that DNA strand is potentially broken in locations closer to 3′ LIRs with larger stem lengths. In addition, a positive moderately significant correlation was found between deletion size and internal spacer length of 3′ LIRs (*r* = 0.29, *P* < 0.02), with no correlation between internal spacer length of 5′ LIRs (*r* = −0.16, *P* > 0.1). These results show that 3′ LIRs with bigger loops are more important than 5′ LIRs, for larger gross deletions in human genome.

Similarly, associations between deletion size and stem identities of 5′ (*r* = −0.40, *P* < 0.005) and 3′ (*r* = 0.30, *P* < 0.05) LIRs were found in 49 gross deletions including LIR on the both of 5′ and 3′ breakpoints. These data suggest that 3′ LIRs with greater stem identities cause larger deletion sizes, while similar 5′ LIRs cause smaller deletion sizes. Furthermore, a association between distance from breakpoint of 5′ LIRs and stem identity of 3′ LIRs (*r* = 0.28, *P* < 0.05) was also found, suggesting 3′ LIRs with greater stem identities are more likely to induce DNA breakage than 5′ LIRs.

**Figure 7:**
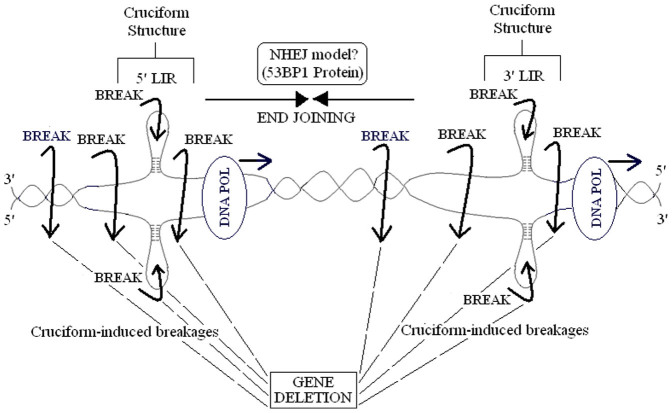
A model mechanism for 5′ and 3′ LIRs-mediated gene deletion. Cruciform structures of LIRs are formed on DNA strands during replication, with breaks potentially occurring inside LIR or near locations. LIR-induced breakages at the 5′ and 3′ breakpoint sequences may cause gene deletion by enabling free DNA ends to recombine via 53BP1-mediated NHEJ. Abbreviations: LIR, long inverted repeat; NHEJ, non-homologous end-joining.

Consequently, LIRs may induce DNA breakages at the nearby locations through forming cruciform structures. Free DNA ends between distant sites may come together by NHEJ, with following gene deletion (Figure 7). Similarly, Varga and Aplan reported that DNA breaks produced various deletions exhibiting NHEJ features in the human monocytic cell line, U937^99^. They showed that aberrant double-strand break repair by NHEJ may lead to gross chromosomal rearrangements including interstitial deletion and large insertions.

In 40 gross deletions containing 5′ or 3′ LIR, positive moderate correlation between internal spacer length and deletion size (*r* = 0.35, *P* < 0.05) was found, similar to the group that included 138 LIRs. In addition, in 24 of 40 gross deletions, new LIRs between distant free ends containing LIR and no LIR were detected (Figures 4 and 5). These results show that LIRs with bigger loops cause larger deletions in human genome, suggesting that larger loops may give rise to greater stress and transition activity on the DNA strand during replication. Moreover, it was reported that bigger inverted repeats can dominate strand separation and B-Z transition, with Zhabinskaya and Benham, showing that long IRs occupy clinically important chromosomal breakpoints corresponded closely with translocation frequencies through probably cruciform extrusion^100^.

In conclusion, these results suggest that a LIR found in 5′ or 3′ breakpoints, may break DNA strand via cruciform structure and match with homolog sequences in other breakpoint site, resulting in a back-folded stem-loop structure during replication (Figure 6). In this way, DNA breakage may also occur in other breakpoint location containing no LIR. After double-strand breakages are formed at 5′ and 3′ breakpoints, DNA ends between distant sites may combine by NHEJ, with following gene deletion.

As presented in Fig. 6, this model is supported with a study carried out in *Saccharomyces cerevisiae*^101^. In this study, IRs with internal spacer of 21 kb were placed into *Saccharomyces cerevisiae* chromosome. After double-strand break was induced, large dicentric inverted dimers were observed, leading to gross chromosomal rearrangements during anaphase stage. In addition, it has been suggested that p53-binding protein 1 (53BP1) combines free DNA ends between distant sites for NHEJ^102^.

An algorithm such as internal spacer <2 kb, stem copy identity >85% and stem length >30 bp for recombinogenic LIRs in human and other organism genomes was suggested^2^. In the present study, only 35 (25.36%) of 138 LIRs located close to the 5′ and 3′ breakpoints from 89 gross deletions, correspond to this criteria (see Supplementary Table S3 online). However, the present findings indicate that significant relationship between LIR numbers and breakpoint regions of gross gene deletions. There is also a strongly positive correlation between 5′ and 3′ LIR numbers on breakpoint regions. On the other hand, 5′ and 3′ LIRs may have converse effects on deletion size. However, over-LIR intensity on 5′ or 3′ breakpoint locations cause smaller deletion sizes. In addition, this study showed that 3′ LIRs may be more active than 5′ LIRs in deletional and recombinational events. Moreover, internal spacer length affects breakage site and deletion size in the gross deletions. Therefore, the present study suggests necessity of a new algorithm for LIRs in breakpoint regions of gross gene deletions associated with human cancers and inherited genetic diseases.

Consequently, LIRs detected in genomic regions including breakpoint sequences of many gross gene deletions, may lead to cruciform structure formation during DNA replication and break DNA strand. After double-strand breaks occur in 5′ and 3′ breakpoints, gene deletions may be formed by combining free DNA ends with 53BP1 for NHEJ.

## Methods

### Gross gene deletions and breakpoint regions

In total, 109 gross gene deletions involving 63 genes, were obtained from the Human Gene Mutation Database (HGMD)^90^,^91^ (see Supplementary Table S1 online). Base sequences of 5′ and 3′ deletion breakpoints were taken from references 33,34,35,36,37,38,39,40,41,42,43,44,45,46,47,48,49,50,51,52,53,54,55,56,57,58,59,60,61,62,63,64,65,66,67,68,69,70,71,72,73,74,75,76,77,78,79,80,81,82,83,84,85,86,87,88,89 listed in the HGMD^90^, or obtained from the Gross Rearrangement Breakpoint Database (GRaBD)^92^,^93^ (see Supplementary Table S1 online). Sequences of genes associated with deletions were downloaded from NCBI^103^. Gene accession numbers are provided (Table 1). Each deletion breakpoint sequence and corresponding genes were compared using NCBI BLAST^104^, and breakpoint locations matched with related genes (Figure 1a). For each gene deletion, nucleotide positions of 5′ and 3′ breakpoints are shown (Table 1). Sequences (±10 kb) spanning 5′ and 3′ breakpoints of gross gene deletions were included in the deletion group (see Supplementary Table S2 online). In total, 218 breakpoint sequences from 109 gross gene deletions were examined for LIR identification (Figure 1b).

For the control group, the DNA sequences of 68 different genes were downloaded from NCBI^103^ to be selected randomly (see Supplementary Table S2 online). Searching the HGMD^90^ site confirmed that selected control genes were not associated with deletions. Subsequently, 20 kb segments of DNA sequence from each control gene were included in the control group. In total, 220 control sequences were examined for LIR identification.

### LIR identification

Identification of LIRs was performed within genomic regions (including the 218 breakpoint sequences from 109 gross gene deletions of 63 genes, and 220 control sequences from 68 genes) using IRF^94^,^95^ software (Figure 1b). The 2, 3, 5 and 40 (match, mismatch, indel and minimum score) parameters of IRF^94^ were selected for identification.

LIRs with stem length >20 bp, internal spacer of 0–10 kb, stem identity ≥70%, and within ±10 kb fragments flanking each of the 5′ and 3′ breakpoint sequences of human gross gene deletions, or 20 kb segments of control genes, were investigated (Figure 1b). Total LIR numbers were determined (see Supplementary Table S2 online) and statistically compared between control and deletion groups. In addition, associations between LIR numbers on 5′ and 3′ breakpoints and also deletion size were statistically investigated.

Recently, Wang and Leung reported that LIRs with stem length >30 bp, stem identity >85% and internal spacer <2 kb were highly recombinogenic in humans and other organisms^2^. It was also shown that long *Alu* IRs with 75% stem identity caused mild replication blockage in *E. coli*^3^. Thus, LIRs with distance of 0–3 kb from breakpoints, stem length >20 bp, internal spacer of 0–2.5 kb, and stem identity ≥70%, were selected for determining associations between LIR features, distances from breakpoint and deletion size (see Supplementary Table S3 online; Figure 1c). At this stage, if many LIRs were observed in the same breakpoint region, the one which best fits the above criteria was chosen.

In addition, 40 of 109 gross gene deletions containing LIRs in only one of regions flanking 5′ and 3′ breakpoints, were further examined. The capacity to form new LIRs between breakpoints with LIRs and other breakpoint sites (including non LIRs of related deletion regions) was researched using IRF^94^.

For this, 5 kb of DNA sequence from breakpoints containing LIRs, and 10 kb of DNA sequence including other breakpoints but containing no LIRs, were combined before scanning for LIRs using IRF^94^. During this process, deleted gross genes were excluded and combined DNA sequences used. LIRs with stem length >150 bp and >70% stem identity were selected for determining associations between LIR features and distance from breakpoints (see Supplementary Table S4 online).

### Statistical analysis

Mann-Whitney *U* test was used for statistical comparison of mean ranks of LIR numbers between gross gene deletion and control groups. Pearson's (*r*) and Spearman's (*r_s_*) correlation coefficients were used to examine associations between LIR features (stem length and identity, and loop length), and distance from breakpoint and gene deletion size. In addition, Pearson's and Spearman's correlation coefficients were also used for determining associations between deletion size and 5′ and 3′ LIR numbers within ±10 kb sequence spanning each breakpoint in 109 gross deletions. Correlation coefficients (*r*, *r_s_*) were classified according to criteria as low (0.00–0.24), moderate (0.25–0.49), strong (0.50–0.74) and strongly (0.75–1.00)^105^. Two-sided *P* values < 0.05 were considered statistically significant. All analyses were performed using SPSS 11.0 software (Chicago, USA).

## Author Contributions

N.A. conceived the study and performed the bioinformatics methods and analysed the statistical data using SPSS 11.0 software, wrote the manuscript and revised it, and prepared all figures and tables.

## Supplementary Material

Supplementary Information Supplementary information

## Figures and Tables

**Table 1:** 5′ and 3′ breakpoint locations from gross gene deletions and detection of long inverted repeats*

GENE/DELETION SIZE	BREAKPOINT LOCATION/NCBI ACCESSION NUMBER	5′LIR/3′LIR	GENE/DELETION SIZE	BREAKPOINT LOCATION/NCBI ACCESSION NUMBER	5′LIR/3′LIR
1-*BRCA1*/14 kb	(3′) 16223-30238 (5′)/NT_010755.15	+/+	32-*PTEN*/662 kb	(5′) 475951-1138023 (3′)/NT_030059.12	+/−
2-*BRCA1*/3.8 kb	(3′) 59697-63533 (5′)/NT_010755.15	+/+	33-*PTEN*/13.6 kb	(5′) 607242-620845 (3′)/NT_030059.12	+/−
3-*BRCA1*/36.3 kb	(5′) 29197-65577 (3′)/L78833.1	+/+	34-*PTEN*/300 kb	(5′) 612328-912640 (3′)/NT_030059.12	−/−
4-*BRCA1*/19.8 kb	(5′) 51483-71369 (3′)/L78833.1	+/+	35-*MEN1*/67.7 kb	(3′) 131486-199212 (5′)/AC000134.14	+/+
5-*BRCA1*/18.3 kb	(5′) 56990-75331 (3′)/L78833.1	+/+	36-*BTK*/5.9 kb	(5′) 62499-68391 (3′)/U78027.1	+/+
6-*BRCA1*/4.8 kb	(5′) 62115-66940 (3′)/L78833.1	+/+	37-*HBB*/9.1 kb	(5′) 53257-62324 (3′)/U01317.1	+/−
7-*BRCA2*/5.1 kb	(5′) 3806-8872 (3′)/NC_000013.9	+/+	38-*ATM*/3.4 kb	(5′) 73863-77312 (3′)/U82828.1	+/+
8-*BRCA2/*14.3 kb	(5′) 12323-26644 (3′)/AY436640.1	+/−	39-*MSH2*/36.6 kb	(5′) 99957-136583 (3′)/AC079775.6	+/+
9-*BRCA2*/7.9 kb	(5′) 27566-35513 (3′)/AY436640.1	−/+	40-*MSH2*/2.2 kb	(5′) 104712-106890 (3′)/AC079775.6	+/+
10-*BRCA2*/10.8 kb	(5′) 45138-55975 (3′)/AY436640.1	+/+	41-*MSH2*/15.4 kb	(5′) 105900-121280 (3′)/AC079775.6	+/+
11-*BRCA2*/4.9 kb	(5′) 56447-61399 (3′)/AY436640.1	+/+	42-*MSH2*/6.7 kb	(5′) 124511-131175 (3′)/AC079775.6	+/+
12-*CDKN*/33.8 kb *2A/2B*	(3′) 176468-210312 (5′)/NT_008413.17	−/+	43-*MLH1*/6.1 kb	(3′) 86839-92892 (5′)/AC006583.31	+/+
13-*NF1*/99.6 kb	(5′) 238391-338000 (3′)/NT_010799.14	−/+	44-*MLH1*/3.5 kb	(3′) 93192-96718 (5′)/AC006583.31	−/+
14-*NF1*/12 kb	(5′) 347607-359613 (3′)/NT_010799.14	+/−	45-*MLH1*/14.3 kb	(5′) 145570-159901 (3′)/AC011816.17	+/+
15-*RB1*/52.3 kb	(5′) 305422-357770 (3′)/NT_024524.13	−/−	46-*MLH1*/12.7 kb	(5′) 147927-160607 (3′)/AC011816.17	+/+
16-*RB1*/178 kb	(5′) 312336-490300 (3′)/NT_024524.13	+/−	47-*MSH6*/11.3 kb	(5′) 5698337-5709648 (3′)/NT_034483.3	+/+
17-*RB1*/201.9 kb	(5′) 356866-558723 (3′)/NT_024524.13	+/−	48-*MSH6*/21.7 kb	(5′) 5699487-5721160 (3′)/NT_034483.3	+/+
18-*RB1*/40 kb	(5′) 365612-405655 (3′)/NT_024524.13	−/+	49-*PMS2*/1.1 kb	(5′) 53291-54449 (3′)/AC005995.3	+/+
19-*RB1*/3.9 kb	(5′) 378370-382275 (3′)/NT_024524.13	+/−	50-*HEXA*/7.9 kb	(3′) 449987-457933 (5′)/NT_010194.16	−/+
20-*RB1*/2.4 kb	(5′) 462638-465032 (3′)/NT_024524.13	+/−	51-*HPRT1*/13.3 kb	(5′) 297453-310733 (3′)/NT_011786.15	+/+
21-*APC*/435 kb	(5′) 384224-819689 (3′)/NT_034772.5	−/+	52-*HPRT1*/159 bp	(5′) 314410-314569 (3′)/NT_011786.15	−/−
22-*APC*/737 kb	(5′) 529476-1266975 (3′)/NT_034772.5	−/−	53-*LDLR*/7.1 kb	(5′) 191462-198534 (3′)/NT_011295.10	+/+
23-*TSC2*/8.4 kb	(5′) 417616-426044 (3′)/NT_037887.4	+/−	54-*PAX6*/1002 kb	(3′) 750100-1752166 (5′)/NT_009237.17	−/+
24-*TSC2*/10.1 kb	(5′) 432071-442186 (3′)/NT_037887.4	+/−	55-*PAX6*/836 kb	(3′) 905528-1741689 (5′)/NT_009237.17	−/−
25-*TSC2*/916 bp	(5′) 437300-438216 (3′)/NT_037887.4	+/+	56-*IGHM*/732 kb	(3′) 204471-936631 (5′)/NT_026437.11	−/−
26-*TSC1*/7.6 kb	(3′) 177985-185621 (5′)/NT_035014.4	−/+	57-*PKLR*/1.1 kb	(5′) 9489-10630 (3′)/AY316591.1	+/−
27-*ADA*/3.2 kb	(3′) 500551-503808 (5′)/NT_011362.9	+/+	58-*LHCGR*/6.1 kb	(5′) 63641-69726 (3′)/NG_008193.1	−/+
28-*WT1*/7.3 kb	(3′) 32504-39823 (5′)/NC_000011.8	−/+	59-F 8/19.9 kb	(5′) 105125-125067 (3′)/AY769950.1	+/−
29-DMD/7.8 kb	(3′) 152815-160602 (5′)/NT_011757.15	+/−	60-F 8/29.2 kb	(5′) 29349-58524 (3′)/AY769950.1	−/−
30-DMD/3.8 kb	(3′) 155384-159231 (5′)/NT_011757.15	−/+	61-STS/40.1 kb	(5′) 32594-72652 (3′)/NT_011757.15	−/−
31-PTEN/453 kb	(5′) 452645-905607 (3′)/NT_030059.12	+/+	62-WAS/1.9 kb	(5′) 2932-4803 (3′)/AF115549.2	+/+
63-*VWF*/61 kb	(5′) 20561-81606 (3′)/NG_009072.1	+/+	87-*CFTR*/1.5 kb	(5′) 184647-186177 (3′)/NC_000007.12	−/−
64-*VWF*/2.3 kb	(5′) 146608-148929 (3′)/NG_009072.1	−/+	88-*CFTR*/21.1 kb	(5′) 18350-39431 (3′)/NC_000007.12	+/+
65-*VPS13B*/1.8 kb	(5′) 8039-9822 (3′)/NG_007098.2	+/+	89-*CHRNE*/1.3 kb	(3′) 1335845-1337134(5′)/NT_010823.11	+/−
66-*SMN1*/6.7 kb	(5′) 23935-30628 (3′)/NG_008691.1	+/+	90-*COL17A1/*21 kb	(5′) 11200-32195 (3′)/NG_007069.1	−/−
67-*SLC3A1*/30.3 kb	(5′) 101289-131606 (3′)/AC013717.8	+/+	91-*CYBB*/25.3 kb	(5′) 37250-62577 (3′)/NT_079573.3	−/−
68-*SERPING*/2.9 kb	(5′) 4337-7226 (3′)/X54486.1	+/+	92-*FANCA*/2.1 kb	(3′) 20800-22859 (5′)/NT_010542.15	+/+
69-*SDHC*/8.4 kb	(5′) 85491-93861 (3′)/AL592295.25	+/+	93-*FANCA*/44.1 kb	(3′) 32415-76544 (5′)/NT_010542.15	+/+
70-*SALL4*/8.9 kb	(3′) 23922-32811 (5′)/AL034420.16	+/−	94-*FBN1*/6.4 kb	(5′) 187965-194400 (3′)/NG_008805.1	−/−
71-*PROS1*/4.4 kb	(3′) 89888-94322 (5′)/NC_000003.10	+/+	95-*FBN1*/7.1 kb	(5′) 197759-204893 (3′)/NG_008805.1	−/+
72-*PREPL*/23.9 kb	(3′) 120275-144148 (5′)/AC013717.8	−/+	96-*FERMT1*/3.9 kb	(5′) 70250-74168 (3′)/AL118505.17	+/+
73-*PREPL*/38.1 kb	(3′) 123903-162024 (5′)/AC013717.8	+/+	97-*FGA*/15.2 kb	(3′) 47644-62873 (5′)/AC107385.4	−/−
74-*PKHD1*/7.3 kb	(5′) 332887-340206 (3′)/AY129465.1	−/−	98-*FOXL2*/8.2 kb	(3′) 60868-69094 (5′)/AC092947.12	−/−
75-*PKHD1*/13.2 kb	(5′) 337078-350281 (3′)/AY129465.1	−/+	99-*GAA*/8.3 kb	(5′) 97036-105323 (3′)/NT_024871.11	−/−
76-*PKD1*/2.9 kb	(5′) 18177-21076 (3′)/L39891.1	−/−	100-*GBE1*/105.7 kb	(3′) 119019-224705 (5′)/NC_000003.10	−/+
77-*PINK1*/4.6 kb	(5′) 18515-23118 (3′)/NG_008164.1	+/+	101-*GHR*/4.1 kb	(5′) 437912-442010 (3′)/NT_006576.15	−/−
78-*PARK2*/156 kb	(5′) 484956-641159 (3′)/NG_008289.1	+/−	102-*GLA*/4.5 kb	(3′) 30733-35251 (5′)/NT_011651.16	+/+
79-*OCA2*/122.6 kb	(3′) 131887-254458 (5′)/NW_925783.1	+/−	103-*GLA*/4.6 kb	(3′) 36143-40797 (5′)/NT_011651.16	+/+
80-*NSD1*/23.9 kb	(5′) 15361-39242 (3′)/NT_023133.12	+/+	104-*GLI3*/176 kb	(3′)15719-53929(5′)/AC005026.2-AC005158.3	+/−
81-*NSD1*/8 kb	(5′) 170994-178991 (3′)/NT_023133.12	+/+	105-*GLI3*/151 kb	(3′) 64730-77461 (5′)/AC005026.2-AC005158.3	−/−
82-*MLC1*/2.1 kb	(5′) 9586-11738 (3′)/NG_009162.1	+/+	106-*GLI3*/1.01 Mb	(3′)154476-127754(5′)/AC012596.4- AC099798.4	−/−
83-*ATP7A*/15.2 kb	(3′) 12883-28093 (5′)/Z94801.1	−/+	107-*GLI3*/728 kb	(3′) 149442-238453 (5′)/AC012596.4-AC005158.3	+/−
84-*ATP7A*/13.7 kb	(3′) 19564-33298 (5′)/Z94801.1	+/+	108-*GLI3*/6.0 Mb	(3′) 7206-147487 (5′)/AC004844.1-AC005483.1	−/+
85-*ATP7A*/13.7 kb	(3′) 31126-44864 (5′)/Z94801.1	+/+	109-*HBA1*/11.2 kb	(5′) (31695-31724)-(42846-42867) (3′)/NG_000006.1	+/+
86-*AVPR2*/21.5 kb	(5′) 54052-75566 (3′)/U52112.2	+/−			

*5′ and 3′ deletion breakpoint sequences were obtained from HGMD and GRaBD. DNA sequences of gene contigs were downloaded from NCBI. DNA sequences of 5′ and 3′ breakpoints and related gene contigs were compared using NCBI-Blast, and breakpoint locations of gross gene deletions determined. LIRs within genomic regions that included gene breakpoint sequences were investigated. Abbreviations: Bp, base pair; GRaBD, gross rearrangement breakpoint database; HGMD, human gene mutation database; kb, kilobase; LIR, long inverted repeat; Mb, megabase.
