# Supplementary material for: Correlations between long inverted repeat (LIR) features, deletion size and distance from breakpoint in human gross gene deletions
Source: Sci Rep. 2015 Feb 6;5:8300. doi: 10.1038/srep08300 (PMC4319165; doi:10.1038/srep08300)
Supplement: Supplementary Information [file srep08300-s1.pdf]

Supplementary information

Correlations between long inverted repeat (LIR) features, deletion size and distance from breakpoint in human gross gene deletions

Nevim Aygun [1,*]

[1] Department of Medical Biology, Faculty of Medicine, Dokuz Eylul University, Inciralti, Izmir, Turkey.

[*]Corresponding author: nevim.aygun@gmail.com

Supplementary Tables

Page No:

Table S1 | Gross gene deletions associated with cancers and genetic diseases, obtained from Human Gene Mutation Database (HGMD) …..………….. 3-4

Table S2 | Deletion sizes and total LIR numbers for deletion and control groups ….. 5-13

Table S3 | Properties of LIRs selected from genomic regions including 5' and 3' breakpoint locations …………………………………………………… 14-15

Table S4 | Properties of new LIRs identified between distant genomic regions including 5' and 3' breakpoint sequences ……………………………….. 16

Supplementary Figures

Page No:

Figure S1 | Distribution of 138 LIRs in the 5' and 3' breakpoint regions from 109 gross gene deletions ………………………………………………….. 17

Figure S2 | Stem lengths of 138 LIRs identified in 5'and/or 3' breakpoints from 89 gross deletions …………………………………………………….... 18

Figure S3 | Stem identities of 138 LIRs identified in 5'and/or 3' breakpoints from 89 gross deletions ……………………………………………………… 19

Figure S4 | Distance from breakpoint of the 138 LIRs located at the 5' and 3' breakpoints of gross gene deletions ……………………………………… 20

Figure S5 | Internal spacer of the 138 LIRs located at the 5' and 3' breakpoints of gross gene deletions ……………………………………………………… 21

Figure S6 | Properties of LIRs that included at both 5' and 3' breakpoint sequences from 49 genomic regions ………………………………………………… 22

Table S1 | Gross gene deletions associated with cancers and genetic diseases, obtained from Human Gene Mutation Database (HGMD)

GENE	PHENOTYPE	REFERENCES [a]	GENE	PHENOTYPE	REFERENCES [a]
1-*BRCA1*	Breast cancer	GRaBD	32-*PTEN*	Cowden disease	Chibon et al.[b, 43]
2-*BRCA1*	Breast cancer	GRaBD	33-*PTEN*	Cowden disease	Chibon et al.[b, 43]
3-*BRCA1*	Breast, ovarian cancer	Montagna et al.[33]	34-*PTEN*	Cowden disease	Chibon et al.[b, 43]
4-*BRCA1*	Breast, ovarian cancer	Tancredi et al.[34]	35-*MEN1*	Multiple endocrin neoplasia 1	Kikuchi et al.[44]
5-*BRCA1*	Breast, ovarian cancer	Agata et al.[35]	36-*BTK*	Agammaglobulinemia	Jo et al.[45]
6-*BRCA1*	Breast, ovarian cancer	Montagna et al.[33]	37-*HBB*	Thalassemia, delta-beta	Palena et al.[46]
7-*BRCA2*	Breast cancer	GRaBD	38-*ATM*	Ataxia telangiectasia	Mitui et al.[47]
8-*BRCA2*	Breast cancer	Agata et al.[36]	39-*MSH2*	Lynch syndrome	Li et al.[48]
9-*BRCA2*	Breast cancer, male	Tournier et al.[37]	40-*MSH2*	Lynch syndrome	Li et al.[48]
10-*BRCA2*	Breast cancer	Agata et al.[36]	41-*MSH2*	Lynch syndrome	Li et al.[48]
11-*BRCA2*	Breast cancer	Agata et al.[36]	42-*MSH2*	Lynch syndrome	Li et al.[48]
12-*CDKN2A/2B*	T-cell ALL	GRaBD	43-*MLH1*	Lynch syndrome	Li et al.[48]
13-*NF1*	Neurofibromatosis 1	Vandenbroucke et al.[38]	44-*MLH1*	Lynch syndrome	Li et al.[48]
14-*NF1*	Neurofibromatosis 1	GRaBD	45-*MLH1*	Lynch syndrome	Li et al.[48]
15-*RB1*	Retinoblastoma	GRaBD	46-*MLH1*	Lynch syndrome	Li et al.[48]
16-*RB1*	Breast cancer	GRaBD	47-*MSH6*	Lynch syndrome	van der Klift et al.[49]
17-*RB1*	Retinoblastoma	GRaBD	48-*MSH6*	Lynch syndrome	van der Klift et al.[49]
18-*RB1*	Osteosarcoma	GRaBD	49-*PMS2*	Lynch syndrome	van der Klift et al.[49]
19-*RB1*	Retinoblastoma	GRaBD	50-*HEXA*	Tay-Sachs disease	Myerowitz et al.[b,50]
20-*RB1*	Retinoblastoma	GRaBD	51-*HPRT1*	Fanconi anemia	GRaBD
21-*APC*	Familial adenomatous polyposis	Takahashi et al.[b, 39]	52-*HPRT1*	Fanconi anemia	GRaBD
22-*APC*	Familial adenomatous polyposis	Takahashi et al.[b, 39]	53-*LDLR*	Hypercholesterolemia	GRaBD
23-*TSC2*	Tuberous sclerosis	Longa et al.[40]	54-*PAX6*	Aniridia	Lauderdale et al.[51]
24-*TSC2*	Tuberous sclerosis	Longa et al.[40]	55-*PAX6*	Aniridia	Lauderdale et al.[51]
25-*TSC2*	Tuberous sclerosis	Longa et al.[40]	56-*IGHM*	Hypogammaglobulinemia	van Zelm et al.[b,52]
26-*TSC1*	Tuberous sclerosis	Longa et al.[40]	57-*PKLR*	Haemolytic anaemia	Baronciani and Beutler[53]
27-*ADA*	Adenosine deaminase deficiency	GRaBD	58-*LHCGR*	Leydig cell hypoplasia	Gromoll et al.[54]
28-*WT1*	Wilms tumour	Tadokoro et al.[41]	59-*F 8*	Haemophilia A	GRaBD
29-*DMD*	Muscular dystrophy, Duchenne.	Gualandi et al.[42]	60-*F 8*	Haemophilia A	Shibata et al.[55]
30-*DMD*	Muscular dystrophy, Duchenne	Gualandi et al.[42]	61-*STS*	Ichthyosis	GRaBD
31-*PTEN*	Cowden disease	Chibon et al.[b, 43]	62-*WAS*	Wiskott-Aldrich syndrome	Imai et al.[56]

Table S1 Continued

GENE	PHENOTYPE	REFERENCES	GENE	PHENOTYPE	REFERENCES
63-*VWF*	Von Willebrand disease 3	Xie et al.[23]	87-*CFTR*	Cystic fibrosis	Audrézet et al.[74]
64-*VWF*	Von Willebrand disease 3	Peake et al.[57]	88-*CFTR*	Cystic fibrosis	Dörk et al.[75]
65-*VPS13B*	Cohen syndrome	Seifert et al.[58]	89-*CHRNE*	Congenital myasthenic syndrome	Abicht et al.[76]
66-*SMN1*	Spinal muscular atrophy	Wirth et al.[b,59]	90-*COL17A1*	Epidermolysis bullosa	Huber et al.[77]
67-*SLC3A1*	Hypotonia-Cystinuria syndrome	Jaeken et al.[60]	91-*CYBB*	Chronic granulomatous disease	GRaBD
68-*SERPING1*	Angioneurotic oedema	Roche et al.[61]	92-*FANCA*	Fanconi anemia	Morgan et al.[78]
69-*SDHC*	Paraganglioma	Baysal et al.[62]	93-*FANCA*	Fanconi anemia	Tipping et al.[79]
70-*SALL4*	Okihiro syndrome	Borozdin et al.[63]	94-*FBN1*	Marfan syndrome	Liu et al.[80]
71-*PROS1*	Protein S deficiency	Schmidel et al.[64]	95-*FBN1*	Marfan syndrome	Liu et al.[80]
72-*PREPL*	Hypotonia-Cystinuria syndrome	Jaeken et al.[60]	96-*FERMT1*	Kindler Syndrome	Has et al.[81]
73-*PREPL*	Hypotonia-Cystinuria syndrome	Jaeken et al.[60]	97-*FGA*	Afibrinogenaemia	Spena et al.[82]
74-*PKHD1*	Polycystic kidney disease	Bergmann et al.[65]	98-*FOXL2*	Blepharophimosis syndrome	Beysen et al.[83]
75-*PKHD1*	Polycystic kidney disease	Bergmann et al.[65]	99-*GAA*	Glycogen storage disease 2	Huie et al.[84]
76-*PKD1*	Polycystic kidney disease 1	Thomas et al.[66]	100-*GBE1*	Glycogen storage disease 4	Tay et al.[85]
77-*PINK1*	Parkinson disease	Li et al.[67]	101-*GHR*	Laron dwarfism	Besson et al.[86]
78-*PARK2*	Parkinsonism	Clarimon et al.[68]	102-*GLA*	Fabry disease	Kornreich et al.[87]
79-*OCA2*	Albinism	Yi et al.[69]	103-*GLA*	Fabry disease	Kornreich et al.[87]
80-*NSD1*	Sotos syndrome	Douglas et al.[70]	104-*GLI3*	Greig cephalopolysyndactyly syndrome.	Johnston et al.[88]
81-*NSD1*	Sotos syndrome	Douglas et al.[70]	105-*GLI3*	Greig cephalopolysyndactyly syndrome	Johnston et al.[88]
82-*MLC1*	Megalencephalic leukoencephalopathy	Leegwater et al.[71]	106-*GLI3*	Greig cephalopolysyndactyly syndrome	Johnston et al.[88]
83-*ATP7A*	Menkes syndrome	Poulsen et al.[72]	107-*GLI3*	Greig cephalopolysyndactyly syndrome	Johnston et al.[88]
84-*ATP7A*	Menkes syndrome	Poulsen et al.[72]	108-*GLI3*	Greig cephalopolysyndactyly syndrome	Johnston et al.[88]
85-*ATP7A*	Menkes syndrome	Poulsen et al.[72]	109-*HBA1*	Thalassaemia alpha	Jia et al.[89]
86-*AVPR2*	Diabetes insipidus, nephrogenic	Schöneberg et al.[73]			

[a] Breakpoint sequences of gross gene deletions were surveyed in references listed in HGMD, although some sequences were obtained from GRaBD.

[b] In addition to HGMD and GRaBD, five references regarding gross gene deletions were from the literature. Abbreviations: GRaBD, gross rearrangement breakpoint database; HGMD, human gene mutation database.

Table S2 | Deletion sizes and total LIR numbers for deletion and control groups*

CONTROL GENE	GENE SIZE (Kb)	SELECTED REGION (Bp)	LIR Nr	DELETION GENE/ BREAK/	DEL SIZE (Bp)	LIR Nr
1-ADAMTS10	30.46	1-20,000	2	1-BRCA1/5'/	14,015	53
2-ACSL4	92.06	1-20,000	20	2-BRCA1/5'/	3,836	23
3-ACSL4	92.06	30,000-50,000	9	3-BRCA1/5'/	36,380	45
4-ACSL4	92.06	60,000-80,000	5	4-BRCA1/5'/	19,886	32
5-ADRA1A	117.26	1-20,000	0	5-BRCA1/5'/	18,341	37
6-ADRA1A	117.26	30,000-50,000	1	6-BRCA1/5'/	4,825	46
7-ADRA1A	117.26	60,000-80,000	2	7-BRCA2/5'/	5,066	10
8-ADRA1A	117.26	90,000-110,000	0	8-BRCA2/5'/	14,321	11
9-BDKRB2	39.53	1-20,000	2	9-BRCA2/5'/	7,947	3
10-BHMT	20.51	1-20,000	8	10-BRCA2/5'/	10,837	18
11-BLNK	79.88	1-20,000	3	11-BRCA2/5'/	4,952	20
12-BLNK	79.88	30,000-50,000	5	12-CDKN2A/2B /5'/	33,844	0
13-BTC	48.44	1-20,000	2	13-NF1/5'/	99,609	2
14-C8A	63.45	1-20,000	1	14-NF1/5'/	12,006	5
15-C8A	63.45	30,000-50,000	11	15-RB1/5'/	52,348	2
16-CRYM	44.58	1-20,000	10	16-RB1/5'/	177,964	1
17-DIAPH1	104.04	1-20,000	10	17-RB1/5'/	201,857	1
18-DIAPH1	104.04	30,000-50,000	2	18-RB1/5'/	40,043	0
19-DIAPH1	104.04	60,000-80,000	12	19-RB1/5'/	3,905	1
20-DKK3	46.38	1-20,000	4	20-RB1/5'/	2,394	3
21-DMGDH	72.02	1-20,000	7	21-APC/5'/	435,465	0
22-DMGDH	72.02	30,000-50,000	2	22-APC/5'/	737,499	3
23-DRD3	50.34	1-20,000	8	23-TSC2/5'/	8,428	12
24-DRD3	50.34	30,000-50,000	9	24-TSC2/5'/	10,115	13
25-ECE1	128.30	1-20,000	6	25-TSC2/5'/	916	13

26-ECE1	128.30	30,000-50,000	9	26-TSC1/5’/	7,636	9
27-ECE1	128.30	60,000-80,000	10	27-ADA/5’/	3,257	12
28-ECE1	128.30	90,000-110,000	22	28-WT1/5’/	7,319	5
29-ELF4	45.79	1-20,000	15	29-DMD/5’/	7,787	4
30-EDARADD	90.33	1-20,000	20	30-DMD/5’/	3,847	4
31-EDARADD	90.33	30,000-50,000	36	31-PTEN/5’/	452,962	5
32-EDARADD	90.33	60,000-80,000	19	32-PTEN/5’/	662,072	7
33-EXO1	41.51	1-20,000	6	33-PTEN/5’/	13,603	5
34-FCRL3	22.67	1-20,000	2	34-PTEN/5’/	300,312	4
35-FREM2	200.10	1-20,000	0	35-MEN1/5’/	67,726	4
36-FREM2	200.10	30,000-50,000	1	36-BTK/5’/	5,892	13
37-FREM2	200.10	60,000-80,000	1	37-HBB/5’/	9,067	1
38-FREM2	200.10	90,000-110,000	1	38-ATM/5’/	3,449	11
39-FREM2	200.10	120,000-140,000	0	39-MSH2/5’/	36,626	48
40-FREM2	200.10	150,000-170,000	3	40-MSH2/5’/	2,178	29
41-FREM2	200.10	180,000-200,000	1	41-MSH2/5’/	15,380	29
42-GAD1	44.46	1-20,000	9	42-MSH2/5’/	6,664	40
43-FMO1	37.45	1-20,000	7	43-MLH1/5’/	6,053	6
44-GARS	39.47	1-20,000	0	44-MLH1/5’/	3,526	5
45-GDNF	24,03	1-20,000	0	45-MLH1/5’/	14,331	5
46-GLTSCR1	95.08	1-20,000	20	46-MLH1/5’/	12,680	7
47-GLTSCR1	95.08	30,000-50,000	32	47-MSH6/5’/	11,311	29
48-GLTSCR1	95.08	60,000-80,000	8	48-MSH6/5’/	21,673	25
49-GOLGA5	45.66	1-20,000	8	49-PMS2/5’/	1,158	58
50-HCRTR2	108.35	1-20,000	2	50-HEXA/5’/	7,946	15
51-HCRTR2	108.35	30,000-50,000	1	51-HPRT1/5’/	13,280	11
52-HCRTR2	108.35	60,000-80,000	6	52-HPRT1/5’/	159	11

53-HK2	60.70	1-20,000	5	53-LDLR/5’/	7,072	75
54-HK2	60.70	30,000-50,000	0	54-PAX6/5’/	1,002,066	1
55-GRB10	203.40	1-20,000	1	55-PAX6/5’/	836,161	3
56-GRB10	203.40	30,000-50,000	0	56-IGHM/5’/	732,160	0
57-GRB10	203.40	60,000-80,000	1	57-PKLR/5’/	1,141	1
58-GRB10	203.40	90,000-110,000	0	58-LHCGR/5’/	6,085	1
59-GRB10	203.40	120,000-140,000	0	59-F 8/5’/	19,942	2
60-GRB10	203.40	150,000-170,000	0	60-F 8/5’/	29,175	4
61-GRB10	203.40	180,000-200,000	0	61-STS/5’/	40,058	1
62-HIBCH	115.20	1-20,000	11	62-WAS/5’/	1,871	9
63-HIBCH	115.20	30,000-50,000	6	63-VWF/5’/	61,045	25
64-HIBCH	115.20	60,000-80,000	3	64-VWF/5’/	2,321	3
65-HIBCH	115.20	90,000-110,000	0	65-VPS13B/5’/	1,783	12
66-IKBKAP	66.81	1-20,000	2	66-SMN1/5’/	6,693	43
67-IKBKAP	66.81	30,000-50,000	0	67-SLC3A1/5’/	30,317	7
68-IMPA2	49.42	1-20,000	4	68-SERPING1/5’/	2,889	14
69-KIF1B	170.90	1-20,000	31	69-SDHC/5’/	8,370	24
70-KIF1B	170.90	30,000-50,000	37	70-SALL4/5’/	8,889	28
71-KIF1B	170.90	60,000-80,000	14	71-PROS1/5’/	4,434	5
72-KIF1B	170.90	90,000-110,000	17	72-PREPL/5’/	23,873	2
73-KIF1B	170.90	120,000-140,000	24	73-PREPL/5’/	38,121	10
74-KIF1B	170.90	150,000-170,000	29	74-PKHD1/5’/	7,319	0
75-LIG1	54.86	1-20,000	20	75-PKHD1/5’/	13,203	0
76-LIG1	54.86	30,000-50,000	11	76-PKD1/5’/	2,899	0
77-KRAS	45.68	1-20,000	3	77-PINK1/5’/	4,603	14
78-LDB3	67.62	1-20,000	3	78-PARK2/5’/	156,203	2
79-LDB3	67.62	30,000-50,000	6	79-OCA2 /5’/	122,571	3

80-LRP8	82.61	1-20,000	0	80-NSD1/5'/	23,881	26
81-LRP8	82.61	30,000-50,000	0	81-NSD1/5'/	7,997	21
82-LRP8	82.61	60,000-80,000	1	82-MLC1/5'/	2,152	12
83-LITAF	39.75	1-20,000	34	83-ATP7A/5'/	15,210	8
84-LRCH1	190.43	1-20,000	0	84-ATP7A/5'/	13,734	11
85-LRCH1	190.43	30,000-50,000	5	85-ATP7A/5'/	13,738	14
86-LRCH1	190.43	60,000-80,000	2	86-AVPR2/5'/	21,514	14
87-LRCH1	190.43	90,000-110,000	13	87-CFTR/5'/	1,530	0
88-LRCH1	190.43	120,000-140,000	2	88-CFTR/5'/	21,081	1
89-LRCH1	190.43	150,000-170,000	0	89-CHRNE/5'/	1,289	36
90-MASTL	32.13	1-20,000	27	90-COL17A1/5'/	20,995	0
91-MFI2	26.53	1-20,000	3	91-CYBB/5'/	25,327	4
92-MCM3AP	50.19	1-20,000	29	92-FANCA/5'/	2,059	36
93-MCM3AP	50.19	30,000-50,000	12	93-FANCA/5'/	44,129	34
94-MYOT	20.0	1-20,000	13	94-FBN1/5'/	6,435	1
95-NLGN3	26.37	1-20,000	11	95-FBN1/5'/	7,134	5
96-NMU	41.07	1-20,000	2	96-FERMT1/5'/	3,918	12
97-OPRM1	236.37	1-20,000	1	97-FGA/5'/	15,229	1
98-OPRM1	236.37	30,000-50,000	3	98-FOXL2/5'/	8,226	2
99-OPRM1	236.37	60,000-80,000	6	99-GAA/5'/	8,287	2
100-OPRM1	236.37	90,000-110,000	1	100-GBE1/5'/	105,686	2
101-OPRM1	236.37	120,000-140,000	1	101-GHR/5'/	4,098	1
102-OPRM1	236.37	150,000-170,000	1	102-GLA/5'/	4,518	22
103-OPRM1	236.37	180,000-200,000	1	103-GLA/5'/	4,654	12
104-OPRM1	236.37	210,000-230,000	5	104-GLI3/5'/	176,334	13
105-OTOA	82.22	1-20,000	22	105-GLI3/5'/	150,856	0
106-OTOA	82.22	30,000-50,000	19	106-GLI3/5'/	1,006,671	0

107-OTOA	82.22	60,000-80,000	10	107-GLI3/5’/	727,747	4
108-PALLD	431.39	1-20,000	2	108-GLI3/5’/	5,952,284	0
109-PALLD	431.39	30,000-50,000	7	109-HBA1/5’/	11,172	5
110-PALLD	431.39	60,000-80,000	2	110-BRCA1/3’/	14,015	15
111-PALLD	431.39	90,000-110,000	8	111-BRCA1/3’/	3,836	37
112-PALLD	431.39	120,000-140,000	2	112-BRCA1/3’/	36,380	53
113-PALLD	431.39	150,000-170,000	0	113-BRCA1/3’/	19,886	52
114-PALLD	431.39	180,000-200,000	1	114-BRCA1/3’/	18,341	55
115-PALLD	431.39	210,000-230,000	1	115-BRCA1/3’/	4,825	51
116-PALLD	431.39	240,000-260,000	1	116-BRCA2/3’/	5,066	9
117-PALLD	431.39	270,000-290,000	3	117-BRCA2/3’/	14,321	4
118-PALLD	431.39	300,000-320,000	14	118-BRCA2/3’/	7,947	5
119-PALLD	431.39	330,000-350,000	1	119-BRCA2/3’/	10,837	20
120-PALLD	431.39	360,000-380,000	4	120-BRCA2/3’/	4,952	17
121-PALLD	431.39	390,000-410,000	9	121-CDKN2A/2B/3’/	33,844	10
122-PDSS1	49.13	1-20,000	19	122-NF1/3’/	99,609	11
123-PSPH	40.53	1-20,000	78	123-NF1/3’/	12,006	4
124-RAF1	80.60	1-20,000	30	124-RB1/3’/	52,348	1
125-RAF1	80.60	30,000-50,000	38	125-RB1/3’/	177,964	4
126-RAF1	80.60	60,000-80,000	9	126-RB1/3’/	201,857	0
127-RIMS1	516.20	1-20,000	0	127-RB1/3’/	40,043	3
128-RIMS1	516.20	30,000-50,000	2	128-RB1/3’/	3,905	4
129-RIMS1	516.20	60,000-80,000	0	129-RB1/3’/	2,394	3
130-RIMS1	516.20	90,000-110,000	3	130-APC/3’/	435,465	23
131-RIMS1	516.20	120,000-140,000	0	131-APC/3’/	737,499	7
132-RIMS1	516.20	150,000-170,000	1	132-TSC2/3’/	8,428	15
133-RIMS1	516.20	180,000-200,000	1	133-TSC2/3’/	10,115	9

134-RIMS1	516.20	210,000-230,000	2	134-TSC2/3’/	916	13
135-RIMS1	516.20	240,000-260,000	1	135-TSC1/3’/	7,636	4
136-RIMS1	516.20	270,000-290,000	2	136-ADA/3’/	3,257	12
137-RIMS1	516.20	300,000-320,000	0	137-WT1/3’/	7,319	9
138-RIMS1	516.20	330,000-350,000	1	138-DMD/3’/	7,787	4
139-RIMS1	516.20	360,000-380,000	1	139-DMD/3’/	3,847	4
140-RIMS1	516.20	390,000-410,000	1	140-PTEN/3’/	452,962	5
141-RIMS1	516.20	420,000-440,000	1	141-PTEN/3’/	662,072	2
142-RIMS1	516.20	450,000-470,000	2	142-PTEN/3’/	13,603	0
143-RIMS1	516.20	480,000-500,000	1	143-PTEN/3’/	300,312	5
144-SNCAIP	151.98	1-20,000	0	144-MEN1/3’/	67,726	22
145-SNCAIP	151.98	30,000-50,000	0	145-BTK/3’/	5,892	10
146-SNCAIP	151.98	60,000-80,000	0	146-HBB/3’/	9,067	1
147-SNCAIP	151.98	90,000-110,000	0	147-ATM/3’/	3,449	13
148-SNCAIP	151.98	120,000-140,000	5	148-MSH2/3’/	36,626	30
149-SSH1	70.38	1-20,000	11	149-MSH2/3’/	2,178	33
150-SSH1	70.38	30,000-50,000	17	150-MSH2/3’/	15,380	42
151-SYT14	226.10	1-20,000	0	151-MSH2/3’/	6,664	32
152-SYT14	226.10	30,000-50,000	3	152-MLH1/3’/	6,053	4
153-SYT14	226.10	60,000-80,000	0	153-MLH1/3’/	3,526	6
154-SYT14	226.10	90,000-110,000	0	154-MLH1/3’/	14,331	17
155-SYT14	226.10	120,000-140,000	0	155-MLH1/3’/	12,680	15
156-SYT14	226.10	150,000-170,000	5	156-MSH6/3’/	11,311	30
157-SYT14	226.10	180,000-200,000	1	157-MSH6/3’/	21,673	32
158-TBC1D1	248.08	1-20,000	8	158-PMS2/3’/	1,158	58
159-TBC1D1	248.08	30,000-50,000	4	159-HEXA/3’/	7,946	6
160-TBC1D1	248.08	60,000-80,000	5	160-HPRT1/3’/	13,280	10

161-TBC1D1	248.08	90,000-110,000	3	161-HPRT1/3’/	159	11
162-TBC1D1	248.08	120,000-140,000	2	162-LDLR/3’/	7,072	70
163-TBC1D1	248.08	150,000-170,000	6	163-PAX6/3’/	1,002,066	2
164-TBC1D1	248.08	180,000-200,000	0	164-PAX6/3’/	836,161	0
165-TBC1D1	248.08	210,000-230,000	2	165-IGHM/3’/	732,160	0
166-TDP1	88.86	1-20,000	0	166-PKLR/3’/	1,141	1
167-TDP1	88.86	30,000-50,000	2	167-LHCGR/3’/	6,085	3
168-TDP1	88.86	60,000-80,000	3	168-F 8/3’/	19,942	0
169-TMPO	33.33	1-20,000	31	169-F 8/3’/	29,175	2
170-TOP1MT	25.53	1-20,000	4	170-STS/3’/	40,058	1
171-UGT1A3	44.17	1-20,000	1	171-WAS/3’/	1,871	9
172-VCAN	110.63	1-20,000	0	172-VWF/3’/	61,045	6
173-VCAN	110.63	30,000-50,000	3	173-VWF/3’/	2,321	3
174-VCAN	110.63	60,000-80,000	0	174-VPS13B/3’/	1,783	12
175-VCAN	110.63	90,000-110,000	0	175-SMN1/3’/	6,693	38
176-WNT3	54.23	1-20,000	16	176-SLC3A1/3’/	30,317	8
177-WNT3	54.23	30,000-50,000	3	177-SERPING1/3’/	2,889	13
178-USP24	148.73	1-20,000	1	178-SDHC/3’/	8,370	17
179-USP24	148.73	30,000-50,000	1	179-SALL4/3’/	8,889	8
180-USP24	148.73	60,000-80,000	0	180-PROS1/3’/	4,434	9
181-USP24	148.73	90,000-110,000	1	181-PREPL/3’/	23,873	6
182-USP24	148.73	120,000-140,000	1	182-PREPL/3’/	38,121	6
183-XPNPEP2	30.58	1-20,000	3	183-PKHD1/3’/	7,319	2
184-XYLT1	368.56	1-20,000	1	184-PKHD1/3’/	13,203	2
185-XYLT1	368.56	30,000-50,000	6	185-PKD1/3’/	2,899	0
186-XYLT1	368.56	60,000-80,000	9	186-PINK1/3’/	4,603	13
187-XYLT1	368.56	90,000-110,000	0	187-PARK2/3’/	156,203	0

188-XYLT1	368.56	120,000-140,000	12	188-OCA2/3’/	122,571	0
189-XYLT1	368.56	150,000-170,000	6	189-NSD1/3’/	23,881	64
190-XYLT1	368.56	180,000-200,000	6	190-NSD1/3’/	7,997	12
191-XYLT1	368.56	210,000-230,000	1	191-MLC1/3’/	2,152	14
192-XYLT1	368.56	240,000-260,000	7	192-ATP7A/3’/	15,210	6
193-XYLT1	368.56	270,000-290,000	7	193-ATP7A/3’/	13,734	8
194-XYLT1	368.56	300,000-320,000	3	194-ATP7A/3’/	13,738	10
195-XYLT1	368.56	330,000-350,000	0	195-AVPR2/3’/	21,514	2
196-WWC1	177.67	1-20,000	6	196-CFTR/3’/	1,530	0
197-WWC1	177.67	30,000-50,000	7	197-CFTR/3’/	21,081	6
198-WWC1	177.67	60,000-80,000	5	198-CHRNE/3’/	1,289	36
199-WWC1	177.67	90,000-110,000	10	199-COL17A1/3’/	20,995	0
200-WWC1	177.67	120,000-140,000	6	200-CYBB/3’/	25,327	2
201-WWC1	177.67	150,000-170,000	11	201-FANCA/3’/	2,059	30
202-XIST	32.09	1-20,000	0	202-FANCA/3’/	44,129	31
203-ZC3H3	103.80	1-20,000	4	203-FBN1/3’/	6,435	0
204-ZC3H3	103.80	30,000-50,000	0	204-FBN1/3’/	7,134	5
205-ZC3H3	103.80	60,000-80,000	0	205-FERMT1/3’/	3,918	9
206-HMCN1	456.40	1-20,000	0	206-FGA/3’/	15,229	4
207-HMCN1	456.40	30,000-50,000	0	207-FOXL2/3’/	8,226	1
208-HMCN1	456.40	60,000-80,000	0	208-GAA/3’/	8,287	6
209-HMCN1	456.40	90,000-110,000	1	209-GBE1/3’/	105,686	1
210-HMCN1	456.40	120,000-140,000	1	210-GHR/3’/	4,098	1
211-HMCN1	456.40	150,000-170,000	1	211-GLA/3’/	4,518	22
212-HMCN1	456.40	180,000-200,000	0	212-GLA/3’/	4,654	21
213-HMCN1	456.40	210,000-230,000	0	213-GLI3/3’/	176,334	2
214-HMCN1	456.40	240,000-260,000	1	214-GLI3/3’/	150,856	0

215-HMCN1	456.40	270,000-290,000	12	215-GLI3/3'/	1,006,671	1
216-HMCN1	456.40	300,000-320,000	0	216-GLI3/3'/	727,747	2
217-HMCN1	456.40	330,000-350,000	0	217-GLI3/3'/	5,952,284	1
218-HMCN1	456.40	360,000-380,000	0	218-HBA1/3'/	11,172	9
219-HMCN1	456.40	390,000-410,000	0	SUM		2,723
220-HMCN1	456.40	420,000-440,000	0			
SUM			1,345			

*Total LIR numbers were determined in 220 control and 218 deletion sequences (for control and deletion groups, respectively) using IRF software. LIRs were detected within ± 10 kb flanking 5' and 3' breakpoint sequences of gene deletions. Control genes were randomly selected (outlined in Materials and Methods) and LIRs investigated within 20 kb segments of DNA sequence. Total LIR numbers were compared using Mann Whitney *U* test between control and deletion groups.

Abbreviations: Bp, base pair; IRF, inverted repeat finder; Kb, kilobase; LIR, long inverted repeat; Nr, number.

Table S3 | Properties of LIRs selected from genomic regions including 5’ and 3’ breakpoint locations*

GENE	5' BREAKPOINT-LIRs				3’ BREAKPOINT-LIRs				GENE	5' BREAKPOINT-LIRs				3’ BREAKPOINT-LIRs			
	IS[a]	SL[a]	SID[a]	DIS[a]	IS	SL	SID	DIS		IS	SL	SID	DIS	IS	SL	SID	DIS
1-*BRCA1*	1014	301	81.75	Incl.	1549	306	82.37	Incl.	28-*WT1*	-				142	286	82.81	535
2-*BRCA1*	1106	118	82.35	Incl.	1232	175	82.39	Incl.	29-*DMD*	16	28	89.28	1897	-			
3-*BRCA1*	3	82	81.92	989	1465	282	77.97	271	30-*DMD*	-				10	57	76.66	1260
4-*BRCA1*	555	180	84.61	944	513	171	81.50	1902	31-*PTEN*	768	310	71.65	1165	960	100	94.00	Incl.
5-*BRCA1*	174	292	79.05	362	460	603	75.48	Incl.	32-*PTEN*	50	42	85.71	1385	-			
6-*BRCA1*	454	299	84.38	632	449	295	79.59	Incl.	33-*PTEN*	83	220	87.94	Incl.	-			
7-*BRCA2*	1214	291	80.47	558	1630	251	86.82	1198	34-*PTEN*	-				-			
8-*BRCA2*	1630	251	86.43	Incl.	-				35-*MEN1*	1246	410	78.31	Incl.	95	71	94.36	1715
9-*BRCA2*	-				988	321	83.43	713	36-*BTK*	2	54	78.18	904	1292	306	83.44	178
10-*BRCA2*	1144	275	83.15	Incl.	488	328	81.32	1898	37-*HBB*	771	305	80.44	978	-			
11-*BRCA2*	0	30	87.09	869	1446	300	88.37	Incl.	38-*ATM*	1741	279	72.93	Incl.	207	291	75.07	222
12-*CDKN 2A/2B*	-				580	315	80.06	642	39-*MSH2*	673	270	84.55	199	24	38	86.84	459
									40-*MSH2*	24	296	84.28	Incl.	783	289	85.46	843
13-*NF1*	-				402	236	82.71	285	41-*MSH2*	808	291	77.88	Incl.	49	30	93.33	633
14-*NF1*	0	50	98.00	361	-				42-*MSH2*	614	266	81.78	261	1248	269	72.69	1509
15-*RB1*	-				-				43-*MLH1*	1454	276	76.97	950	5	27	100.0	2121
16-*RB1*	480	255	87.25	1442	-				44-*MLH1*	-				2422	168	83.62	401
17-*RB1*	960	973	70.54	1946	-				45-*MLH1*	1692	295	88.88	Incl.	1134	298	86.37	Incl.
18-*RB1*	-				515	139	75.17	996	46-*MLH1*	235	113	76.31	1678	374	310	82.70	Incl.
19-*RB1*	301	305	75.64	1876	-				47-*MSH6*	1360	286	86.45	Incl.	1339	293	80.20	Incl.
20-*RB1*	1166	279	79.28	235	-				48-*MSH6*	572	134	84.55	Incl.	935	299	80.92	Incl.
21-*APC*	-				260	300	83.55	766	49-*PMS2*	1260	296	84.76	Incl.	750	327	77.97	Incl.
22-*APC*	-				-				50-*HEXA*	-				1778	170	77.90	Incl.
23-*TSC2*	132	282	85.56	458	-				51-*HPRT1*	0	30	93.33	468	6	30	86.66	2539
24-*TSC2*	1213	306	82.74	Incl.	-				52-*HPRT1*	-				-			
25-*TSC2*	597	290	85.61	616	597	290	85.61	1532	53-*LDLR*	863	261	86.36	Incl.	1618	294	88.03	Incl.
26-*TSC1*	-				657	300	85.01	2110	54-*PAX6*	-				917	288	79.36	1332
27-*ADA*	1092	109	85.71	Incl.	1853	272	83.57	Incl.	55-*PAX6*	-				-			

Table S3 Continued

GENE	5' BREAKPOINT-LIRs				3’ BREAKPOINT-LIRs				GENE	5' BREAKPOINT-LIRs				3’ BREAKPOINT-LIRs			
	IS	SL	SID	DIS	IS	SL	SID	DIS		IS	SL	SID	DIS	IS	SL	SID	DIS
56-*IGHM*	-				-				83-*ATP7A*	-				258	282	83.91	Incl.
57-*PKLR*	2175	307	81.84	1395	-				84-*ATP7A*	1544	232	84.32	Incl.	93	75	79.22	1681
58-*LHCGR*	-				1551	289	84.64	Incl.	85-*ATP7A*	345	288	87.32	18	367	86	79.31	158
59-*F 8*	7	26	92.30	2296	-				86-*AVPR2*	129	182	84.23	539	-			
60-*F 8*	-				-				87-*CFTR*	-				-			
61-*STS*	-				-				88-*CFTR*	884	316	87.46	1954	843	94	97.87	1332
62-*WAS*	254	259	79.31	1733	15	69	81.42	118	89-*CHRNE*	432	629	75.03	474	-			
63-*VWF*	658	319	86.91	Incl.	1917	290	85.42	403	90-*COL17A1*	-				-			
64-*VWF*	-				1238	270	79.85	692	91-*CYBB*	-				-			
65-*VPS13B*	106	280	85.71	403	318	294	83.38	Incl.	92-*FANCA*	1324	280	80.00	Incl.	1073	286	80.00	2070
66-*SMN1*	331	277	83.27	Incl.	34	170	84.79	Incl.	93-*FANCA*	971	291	81.29	50	1120	366	82.27	Incl.
67-*SLC3A1*	555	128	82.03	Incl.	1801	50	84.61	1720	94-*FBN1*	-				-			
68-*SERPING1*	1874	37	84.61	1918	1447	141	85.10	Incl.	95-*FBN1*	-				0	28	96.42	611
69-*SDHC*	2435	58	83.05	1148	1417	302	83.06	1906	96-*FERMT1*	696	272	81.15	230	30	48	81.63	89
70-*SALL4*	370	313	80.49	2311	-				97-*FGA*	-				-			
71-*PROS1*	1348	245	81.71	Incl.	222	303	73.33	2101	98-*FOXL2*	-				-			
72-*PREPL*	-				124	109	75.22	1801	99-*GAA*	-				-			
73-*PREPL*	419	276	79.56	Incl.	621	284	77.00	230	100-*GBE1*	-				1	37	87.17	794
74-*PKHD1*	-				-				101-*GHR*	-				-			
75-*PKHD1*	-				52	63	80.30	1680	102-*GLA*	1281	282	84.02	1206	1558	276	84.22	Incl.
76-*PKD1*	-				-				103-*GLA*	790	279	80.85	1959	104	297	85.00	Incl.
77-*PINK1*	416	262	83.77	Incl.	1736	292	82.65	1785	104-*GLI3*	141	291	80.87	752	-			
78-*PARK2*	722	275	80.63	1038	-				105-*GLI3*	-				-			
79-*OCA2*	2102	527	74.02	663	-				106-*GLI3*	-				-			
80-*NSD1*	1592	199	82.26	577	24	276	81.33	779	107-*GLI3*	277	24	100.0	1079	-			
81-*NSD1*	690	136	83.33	1847	1067	301	86.09	Incl.	108-*GLI3*	-				2410	256	78.37	1139
82-*MLC1*	1670	104	81.13	Incl.	707	299	84.10	Incl.	109-*HBA1*	725	296	84.94	Incl.	106	32	87.87	1729

*LIRs from genomic regions including 5’ and 3’ breakpoints of gross gene deletions were selected. Abbreviations: [a] DIS, distance from breakpoint (base pair, bp); Incl., including breakpoint; IS, internal spacer (bp); SID, stem identity (%); SL, stem length (bp).

Table S4 | Properties of new LIRs identified between distant genomic regions including 5’ and 3’ breakpoint sequences*

No[#]	GENE	5’LIR/3’LIR[a]	LIRs Between 5’ and 3’ BPS[a]			No[#]	GENE	5’LIR/3’LIR	LIRs Between 5’ and 3’ BPS[a]		
			SL[a]	SID[a]	DIS[a]				SL	SID	DIS
8	*BRCA2*	+/ -	240	82.30	748	44	*MLH1*	-/ +	288	83.90	3285
9	*BRCA2*	-/ +		-		50	*HEXA*	-/ +	292	81.31	3935
12	*CDKN2A/2B*	-/ +	298	73.28	4513	54	*PAX6*	-/ +	298	83.55	4062
13	*NF1*	-/ +		-		57	*PKLR*	+/ -		-	
14	*NF1*	+/ -	301	75.22	574	58	*LHCGR*	-/ +	279	77.85	1747
16	*RB1*	+/ -	249	84.06	2956	59	*F 8*	+/ -		-	
17	*RB1*	+/ -		-		64	*VWF*	-/ +	299	79.08	3855
18	*RB1*	-/ +	293	82.09	1217	70	*SALL4*	+/ -	296	80.65	3829
19	*RB1*	+/ -	300	82.23	9330	72	*PREPL*	-/ +	302	79.93	3440
20	*RB1*	+/ -		-		75	*PKHD1*	-/ +		-	
21	*APC*	-/ +		-		78	*PARK2*	+/ -		-	
23	*TSC2*	+/ -	305	79.28	382	79	*OCA2*	+/ -	1789	70.19	971
24	*TSC2*	+/ -	316	81.34	42	83	*ATP7A*	-/ +	296	86.66	1333
26	*TSC1*	- / +	302	75.49	5852	86	*AVPR2*	+/ -		-	
28	*WT1*	-/ +	282	83.15	3167	89	*CHRNE*	+/ -	292	83.21	1380
29	*DMD*	+/ -		-		95	*FBN1*	-/ +		-	
30	*DMD*	-/ +	195	82.65	2998	100	*GBE1*	-/ +		-	
32	*PTEN*	+/ -		-		104	*GLI3*	+/ -		-	
33	*PTEN*	+/ -	296	81.22	1581	107	*GLI3*	+/ -	285	78.23	4550
37	*HBB*	+/ -	173	83.05	3388	108	*GLI3*	-/ +		-	

*40 gross gene deletions containing LIRs in only one of genomic sequences flanking 5’ and 3’ breakpoints, were obtained from Table S3 and re-examined in terms of the capacity to form new LIRs. Before LIRs were identified using IRF, 5 kb of DNA sequence from breakpoints containing a LIR and 10 kb of DNA sequence from breakpoints containing no LIR were combined. Internal spacers between 5 kb and 10 kb segments were excluded. In total, 24 LIRs were detected. [#]Numbers of genes are as in Table S3. Abbreviations: [a] BPS, breakpoint sequences; DIS, distance from breakpoint with no LIR (bp); LIR, long inverted repeat; SID, stem identity (%); SL, stem lenght (bp).

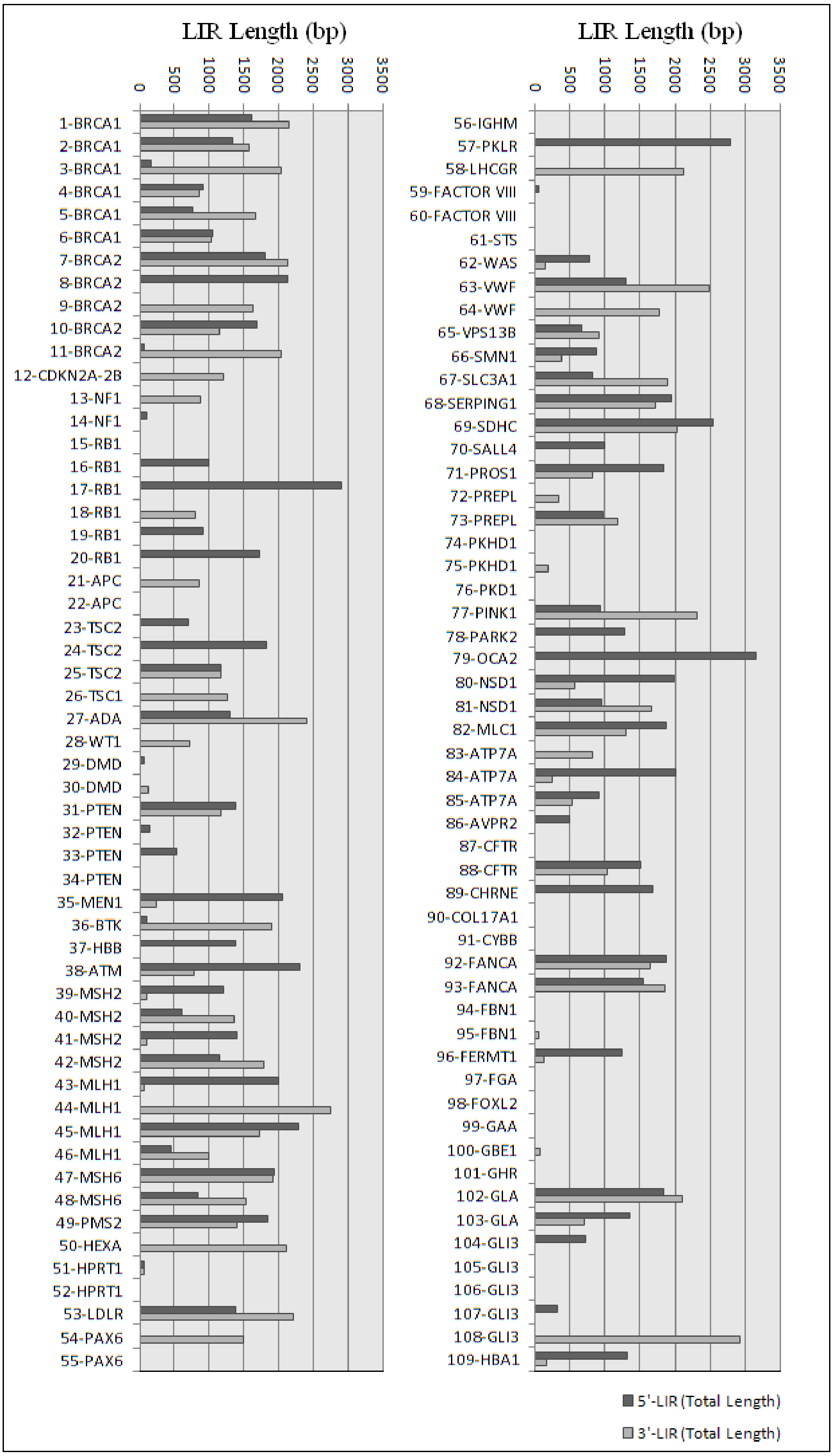

Figure S1 | Distribution of 138 LIRs in the 5' and 3' breakpoint regions from 109 gross gene deletions. Total lengths of each LIR were examined. Total length includes the sum of lengths (bp) of loop and stem copies. Black bars indicate LIRs located at 5' breakpoints, and grey bars LIRs at 3' breakpoints. Abbreviations: bp, base pair; LIR, long inverted repeat.

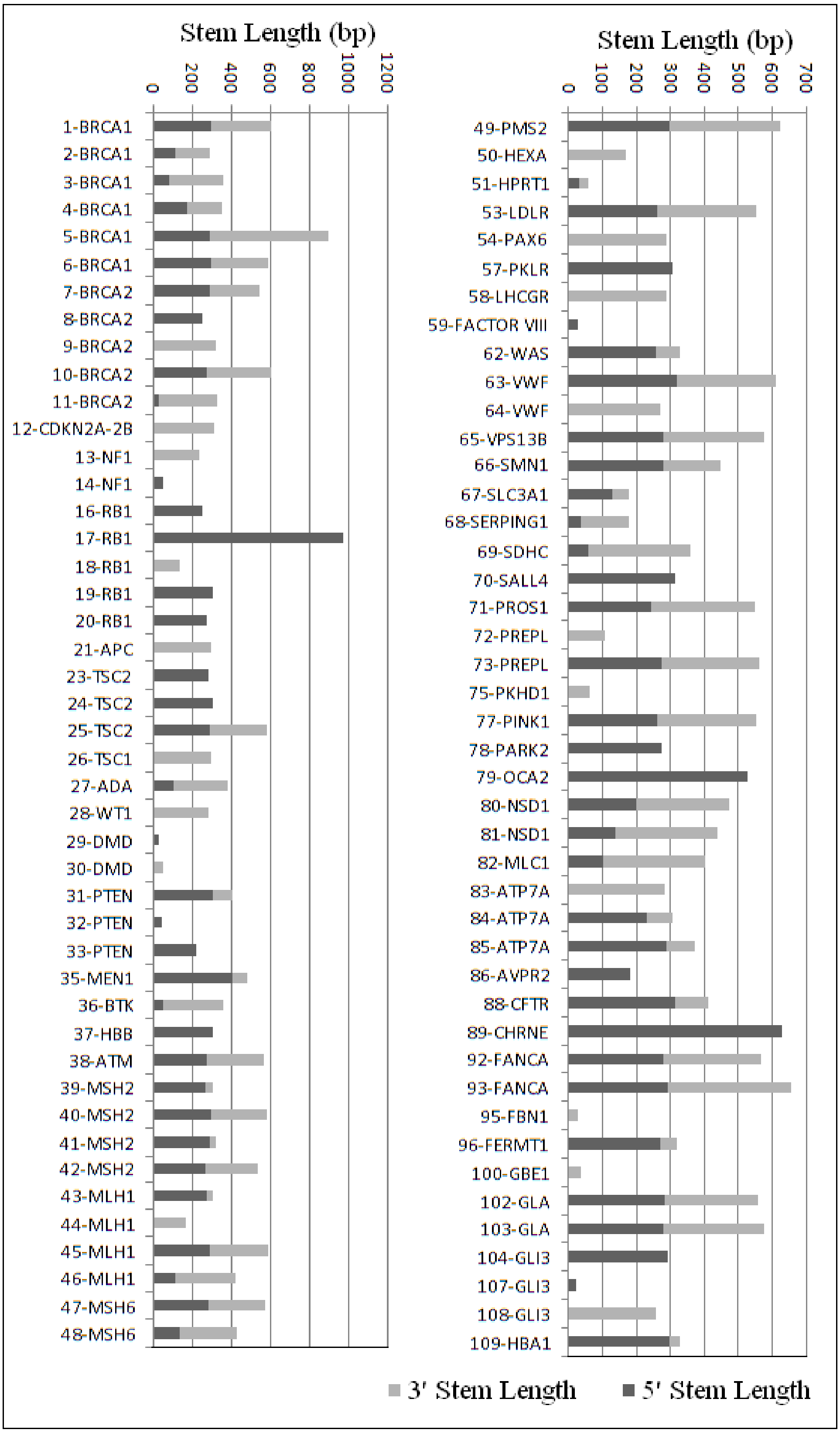

Figure S2│Stem lengths of 138 LIRs identified in 5'and/or 3' breakpoints from 89 gross deletions. Black and grey bars indicate stem lengths of LIRs located at 5' and 3' breakpoints, respectively. Abbreviations: bp, base pair; LIR, long inverted repeat.

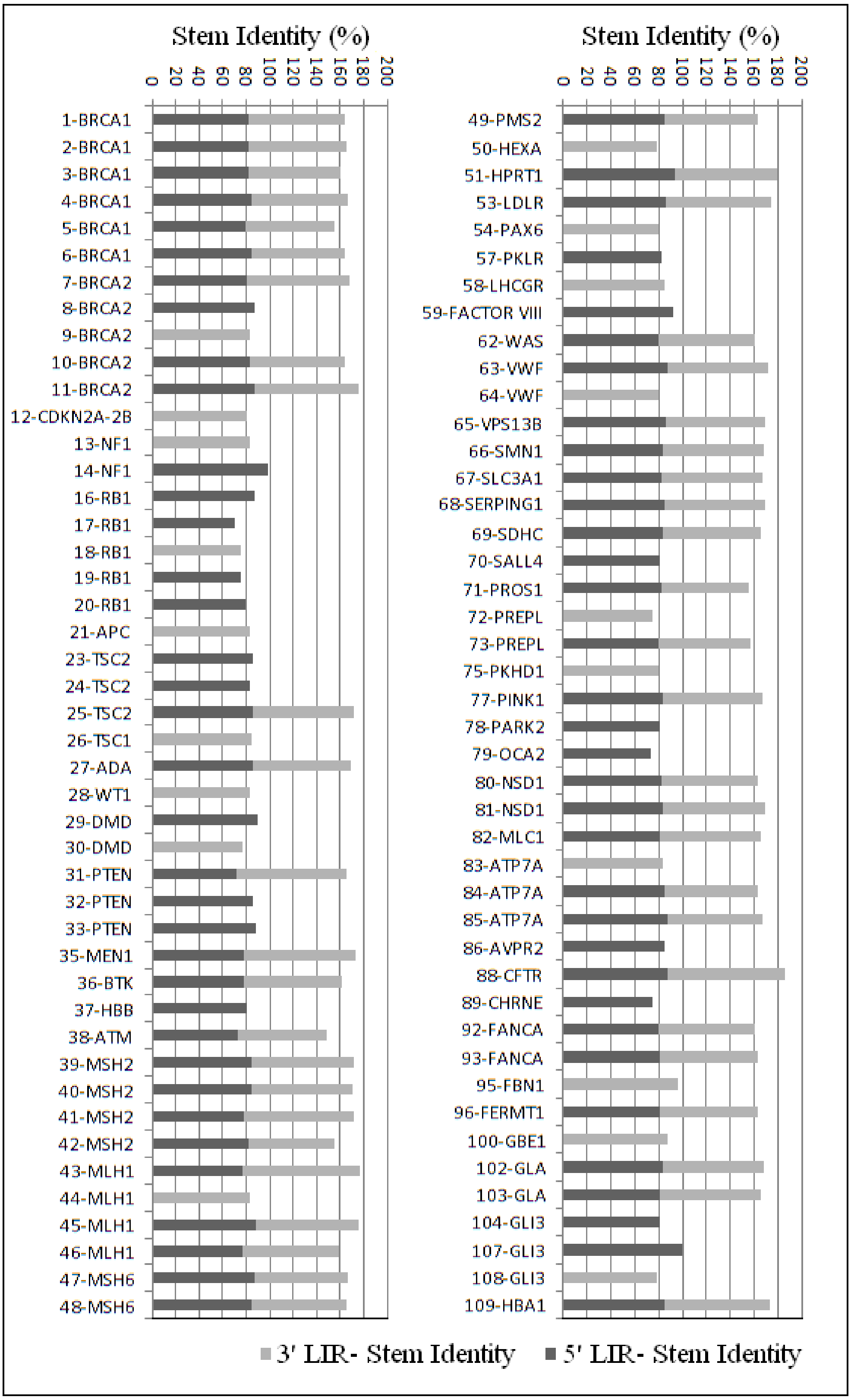

Figure S3│Stem identities of 138 LIRs identified in 5'and/or 3' breakpoints from 89 gross deletions. Black and grey bars indicate stem identities of LIRs located at 5' and 3' breakpoints, respectively. Abbreviations: LIR, long inverted repeat.

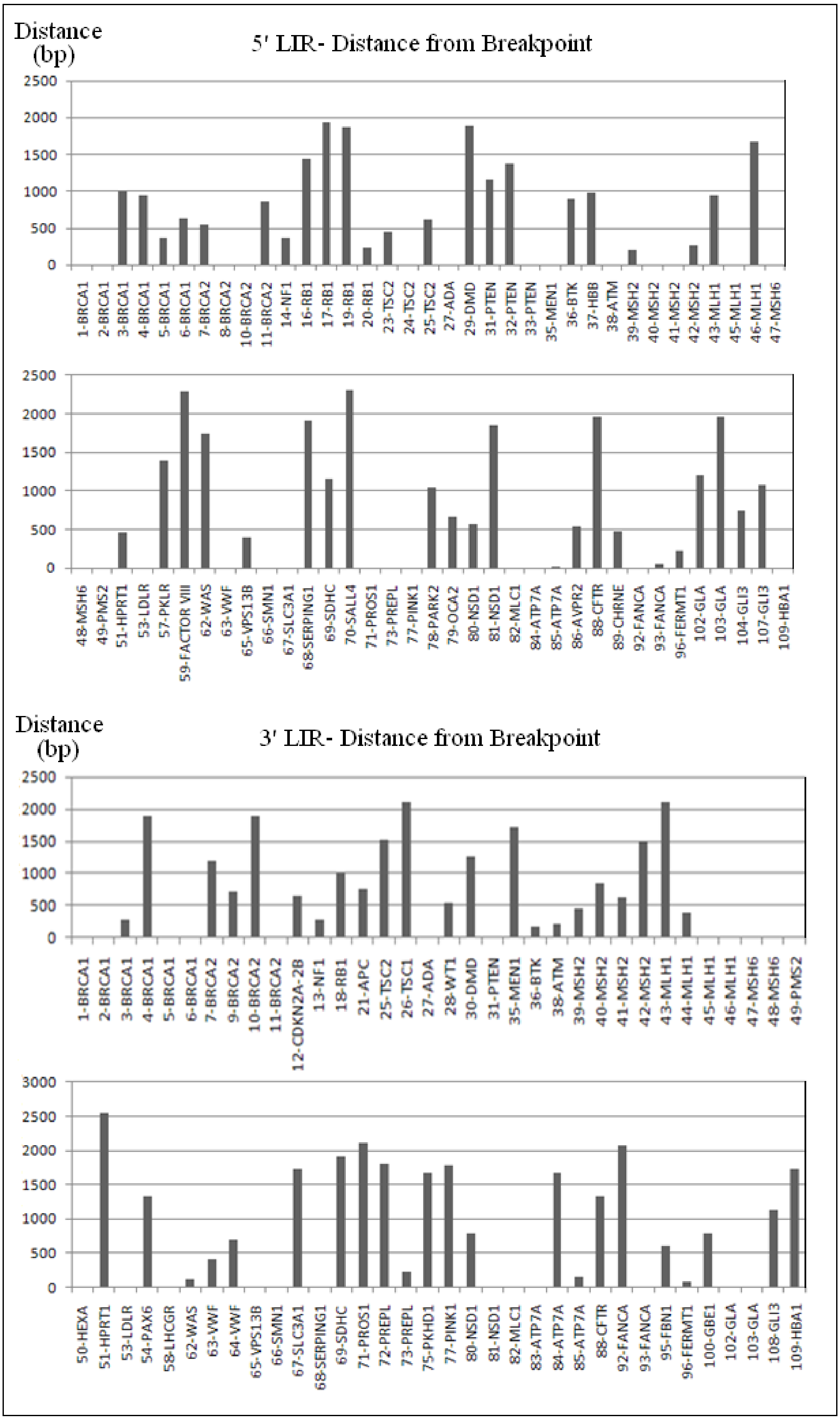

Figure S4 | Distance from breakpoint of the 138 LIRs located at the 5' and 3' breakpoints of gross gene deletions. 70 LIRs were located at ±0–2,311 bp of 5' breakpoint sequences. 68 LIRs were located at ±0–2,539 bp of 3' breakpoint sequences. Abbreviations: bp, base pair; LIR, long inverted repeat.

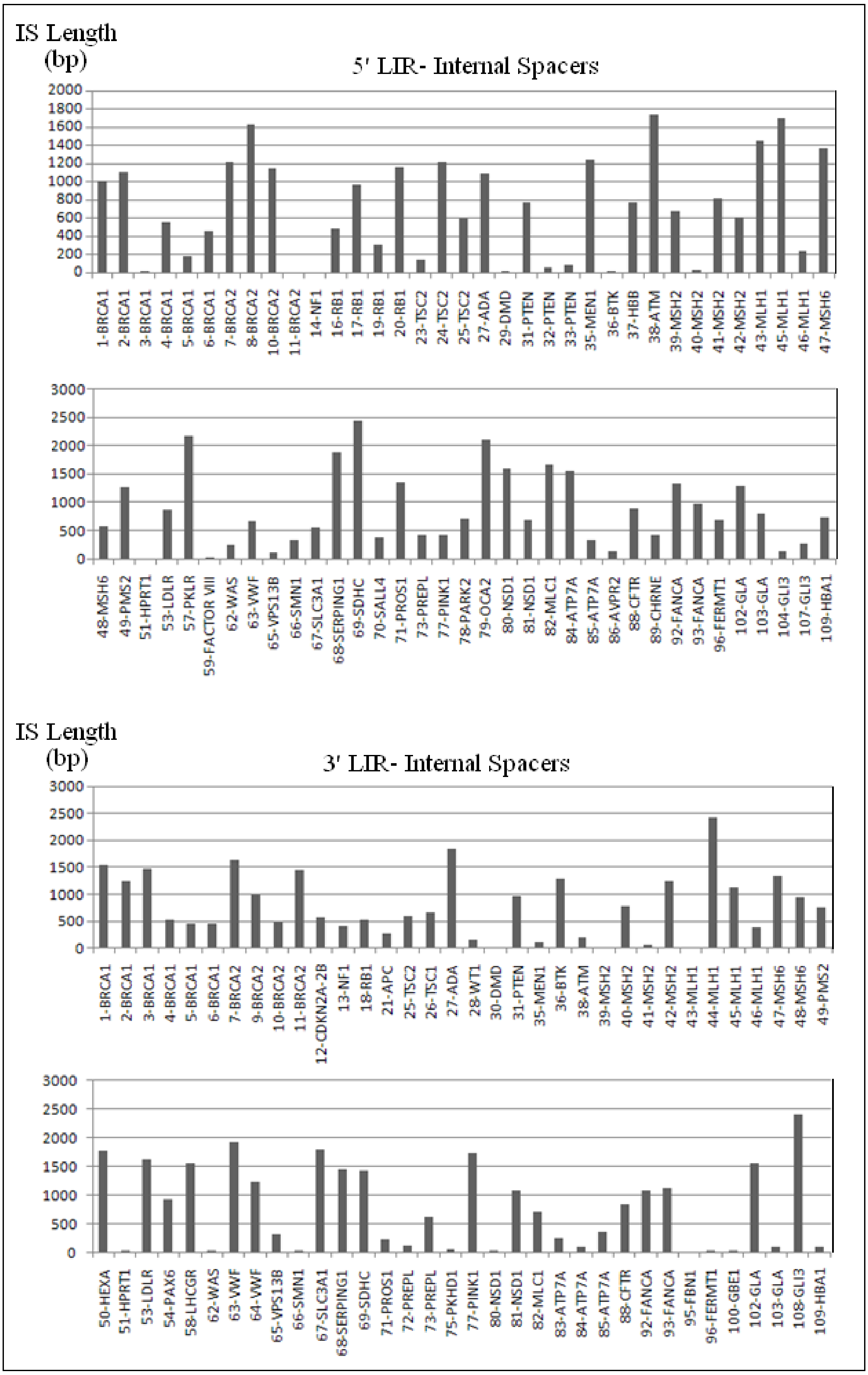

Figure S5 | Internal spacer of the 138 LIRs located at the 5' and 3' breakpoints of gross gene deletions. 70 ISs were between 0 and 2,435 bp in 5' breakpoints. 68 ISs were between 0 and 2,422 bp in 3' breakpoints. Abbreviations: bp, base pair; IS, internal spacer; LIR, long inverted repeat.

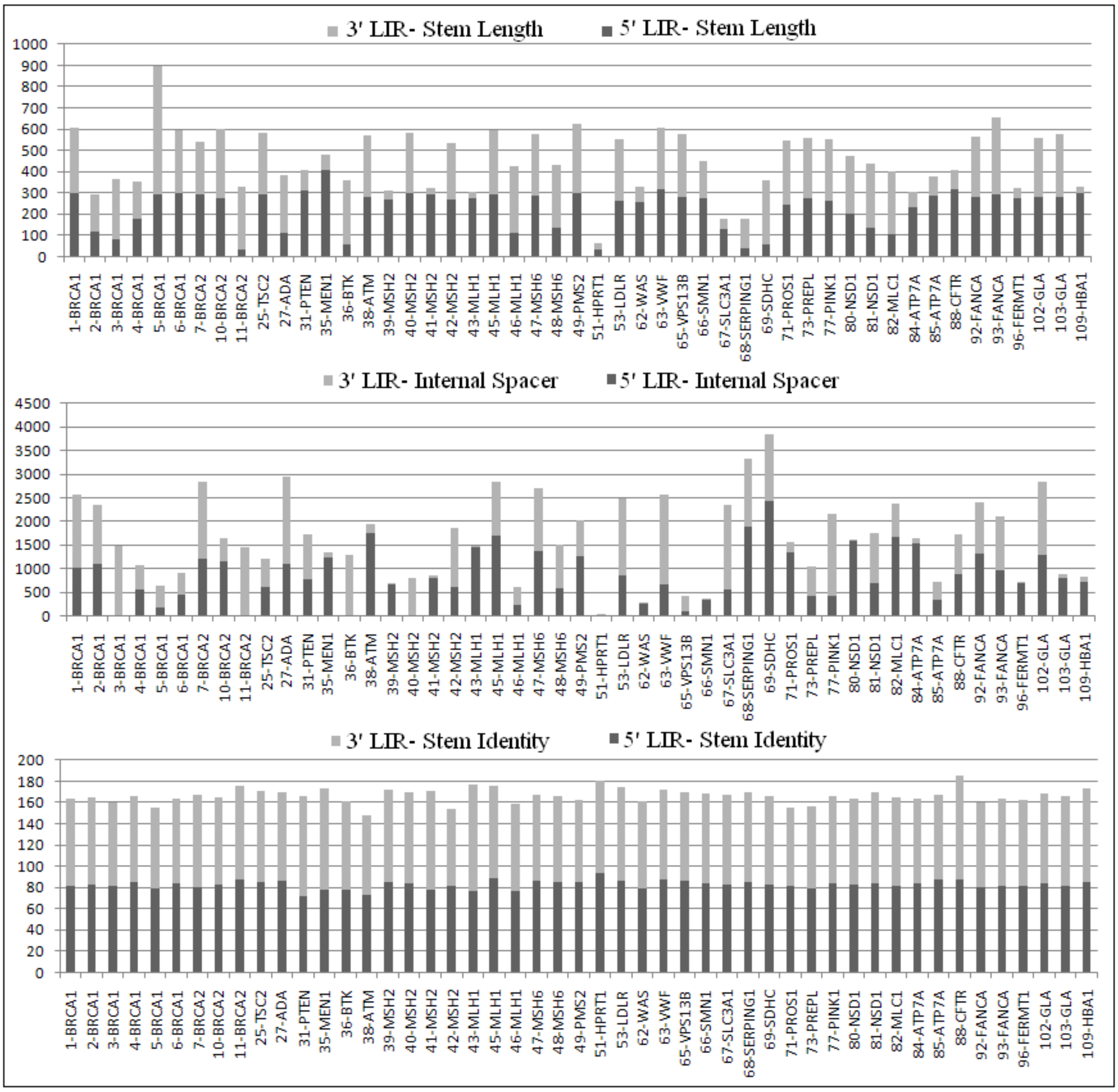

Figure S6│Properties of LIRs that included at both 5' and 3' breakpoint sequences from 49 genomic regions. Total of 98 LIRs detected in both 5' and 3' breakpoints from 49 gross gene deletions. Black and grey bars show stem lengths, internal spacers and stem identities of LIRs located at 5' and 3' breakpoints, respectively. Abbreviations: LIR, long inverted repeat.
